# Manganese Limitation of Phytoplankton Physiology and Productivity in the Southern Ocean

**DOI:** 10.1029/2022GB007382

**Published:** 2022-11-10

**Authors:** Nicholas J. Hawco, Alessandro Tagliabue, Benjamin S. Twining

**Affiliations:** ^1^ Department of Oceanography University of Hawaiʻi at Mānoa Honolulu HI USA; ^2^ School of Environmental Sciences University of Liverpool Liverpool UK; ^3^ Bigelow Laboratory for Ocean Sciences East Boothbay ME USA

**Keywords:** diatoms, photosynthesis, carbon export, subantarctic, trace metals, nutrient limitation

## Abstract

Although iron and light are understood to regulate the Southern Ocean biological carbon pump, observations have also indicated a possible role for manganese. Low concentrations in Southern Ocean surface waters suggest manganese limitation is possible, but its spatial extent remains poorly constrained and direct manganese limitation of the marine carbon cycle has been neglected by ocean models. Here, using available observations, we develop a new global biogeochemical model and find that phytoplankton in over half of the Southern Ocean cannot attain maximal growth rates because of manganese deficiency. Manganese limitation is most extensive in austral spring and depends on phytoplankton traits related to the size of photosynthetic antennae and the inhibition of manganese uptake by high zinc concentrations in Antarctic waters. Importantly, manganese limitation expands under the increased iron supply of past glacial periods, reducing the response of the biological carbon pump. Overall, these model experiments describe a mosaic of controls on Southern Ocean productivity that emerge from the interplay of light, iron, manganese and zinc, shaping the evolution of Antarctic phytoplankton since the opening of the Drake Passage.

## Key Points

- Mn scarcity in the Southern Ocean limits phytoplankton growth in a global biogeochemical model, especially during austral spring
- The spatial extent of Mn limitation is sensitive to phytoplankton traits governing photophysiology and metal homeostasis
- Greater dust deposition to the Southern Ocean expands the role of Mn limitation and restricts carbon export from Fe fertilization

## 1 Introduction

The persistent outgassing of CO_2_ from the Southern Ocean to the atmosphere contributes to the warm interglacial climate of the Holocene (Sarmiento & Toggweiler, 1984; Sigman & Boyle, 2000; Sigman et al., 2010). South of the Polar Front, deep circumpolar water masses upwell into the surface mixed layer, ventilating the deep ocean carbon reservoir and enriching surface waters with high concentrations of the macronutrients nitrate, phosphate, and silicate (Martin, 1990; Sarmiento et al., 2004). The leak of upwelled CO_2_ out of the Southern Ocean can be counteracted by phytoplankton photosynthesis and subsequent carbon export to the deep ocean by the biological carbon pump (Sigman et al., 2010), but only if environmental conditions (light, temperature) permit growth and essential micronutrients, such as iron (Fe), are in sufficient supply (Martínez‐García et al., 2014; Martin, Fitzwater, et al., 1990; Martin, Gordon, et al., 1990).

Outside of the light‐limited winter period, Fe is considered to be the major factor shaping phytoplankton growth in the Southern Ocean spring and summer, with Si limitation of diatoms sometimes occurring in the Subantarctic zone (Boyd, 2002; Tagliabue et al., 2014). The primary production that underpins Southern Ocean ecosystems relies on deep winter mixing to supply dissolved iron (dFe) to the euphotic zone, supplemented by additional sources from dust, hydrothermal vents, continental margins and the cryosphere (Tagliabue et al., 2014, 2017). In this context, increases in Fe supply from dust during glacial periods is postulated to alleviate phytoplankton Fe limitation, enhancing both macronutrient utilization and carbon export in the Southern Ocean and reducing atmospheric CO_2_ (Jaccard et al., 2013; Martin, 1990; Martínez‐García et al., 2014; Sigman et al., 2010).

Phytoplankton primarily need Fe for their photosynthetic apparatus, especially photosystem I (PSI), which contains up to six‐fold more Fe than photosystem II (PSII) (Raven, 1990; Strzepek & Harrison, 2004). Accordingly, many phytoplankton species adapt to Fe limitation with elevated PSII:PSI ratios (by as much as six‐fold), which economizes Fe use (Strzepek & Harrison, 2004). However, recent experiments have shown that high PSII:PSI responses are muted in Southern Ocean lineages (Strzepek et al., 2019), suggesting that other factors supplant the lower Fe requirements afforded by high PSII:PSI. Instead, Fe economy in Southern Ocean phytoplankton is primarily achieved by deploying much larger photosynthetic antennae than temperate species, which reduces the total number of photosystems (PSII and PSI) required for growth (Strzepek et al., 2012, 2019).

The use of Fe in the photosynthetic apparatus occurs alongside manganese (Mn), an essential component of the Mn_4_O_5_Ca oxygen‐evolving complex of PSII (Raven, 1990). Phytoplankton Mn requirements are also driven by the use of Mn as a cofactor in superoxide dismutase, arginase, and other metalloenzymes (Peers & Price, 2004; Twining & Baines, 2013). Dissolved manganese (dMn) is primarily supplied to open ocean surface waters by aeolian deposition and is abundant in the surface of oligotrophic gyres, with typical concentrations ranging from 1 to 5 nM, often an order of magnitude greater than dissolved Fe (dFe) (Boyle et al., 2005; Hatta et al., 2015; Landing & Bruland, 1987). However, phytoplankton uptake of dMn from seawater is complicated by the poor selectivity of their metal transporters for Mn^2+^, the dominant chemical species in seawater, when divalent zinc (Zn), copper (Cu), and cadmium (Cd) ions are present at similar concentrations (Sunda & Huntsman, 1996, 1998b, 2000). This is consistent with predictions from the Irving‐Williams Series, the periodic trend of increasing divalent metal‐binding affinity for organic molecules, following the order Mn^2+^ < Fe^2+^ < Co^2+^ < Ni^2+^ < Cu^2+^ > Zn^2+^ (Irving & Williams, 1953), which shapes metal metabolism and physiology in all domains of life (Waldron & Robinson, 2009). Below the euphotic zone, heterotrophic bacteria oxidize soluble Mn^2+^ to insoluble Mn(III/IV) oxides (Johnson et al., 1996; Sunda & Huntsman, 1988), which accumulate in seafloor sediments. Except for plumes of dMn released from continental margins and hydrothermal vents, Mn oxidation leaves the deep ocean with a low and nearly uniform concentration of dMn, ∼0.3 nM (Hulten et al., 2017).

Deep ocean water masses with low dMn are primarily ventilated in the Southern Ocean, which lacks significant aeolian deposition. Relative to other Fe‐limited regions where Mn supply is greater (e.g., the Subarctic Pacific), the first reports of dMn in the Southern Ocean emphasized unusually low concentrations, proposing the potential for Mn co‐limitation alongside Fe (Martin, Fitzwater, et al., 1990, Martin, Gordon, et al., 1990). More recent surveys have confirmed that dMn in both the Antarctic and Subantarctic zones can be <0.05 nM, the lowest measured globally, especially in remote waters away from continental margin and islands (Balaguer et al., 2022; Browning et al., 2014; Latour et al., 2021; Middag et al., 2011, 2013). Surface waters south of the Polar Front also feature Zn^2+^ concentrations that are 100–1,000‐fold higher than temperate and tropical regions, which should depress algal Mn uptake via competition for membrane transporters (Baars & Croot, 2011; Baars et al., 2014). Indeed, recent experiments have indicated that Mn can be the primary limiting nutrient to phytoplankton growth in the Drake Passage (Browning et al., 2021) and the Ross Sea (Wu et al., 2019), supporting prior suggestions of Fe‐Mn co‐limitation in the Southern Ocean (Browning et al., 2014; Moore, 2016; Martin, Fitzwater, et al., 1990; Martin, Gordon, et al., 1990).

Despite this emerging evidence, the scale of Mn limitation across the Southern Ocean is undefined. The ocean biogeochemistry models that are embedded within Earth System Models have not considered Mn. To date, the only global modeling efforts to simulate Mn biogeochemical cycling have focused on reproducing Atlantic Ocean dMn distributions (Hulten et al., 2017) and the role of zooplankton recycling on several micronutrient elements (Richon & Tagliabue, 2021), but did not address the potential for Mn limitation. To rationalize their experimental results, Browning et al. (2021) applied a simple ecosystem model to show that Mn‐Fe co‐limitation is possible in the Southern Ocean, especially during summer, which agrees with calculations comparing the stoichiometry of Mn, Fe, and macronutrients in phytoplankton and ocean water masses (Moore, 2016). However, these models have not accounted for the range of interactions between phytoplankton physiology, ocean circulation, the carbon cycle and the dynamics of Mn biogeochemistry, including sedimentary inputs from Antarctica and Subantarctic islands, as well as hydrothermal vents. Overall, this limits our understanding of how the low dMn concentrations in Southern Ocean waters can impact phytoplankton growth and the biological carbon pump at large scales, particularly during fluctuations in Fe supply that occur across seasons or during past changes in climate.

Here, we incorporate phytoplankton Mn uptake and Mn requirements into a coupled global ocean physics‐biogeochemistry model to assess the global impact of Mn limitation for the first time. Our simulations explicitly represent a range of mechanistic processes, including external inputs and internal cycling of Mn, alongside the biogeochemical cycles of carbon (C), nitrogen (N), phosphorus (P), silicon (Si), and Fe. We also consider the physiological interactions between light and iron limitation that can influence Mn requirements. Our modeling results highlight a widespread impact of Mn on phytoplankton growth that is most intense during the austral spring and is underpinned by key phytoplankton traits governing light and resource acquisition. Moreover, additional simulations using reconstructions of aeolian dust supply, a source of both Fe and Mn, during the Last Glacial Maximum (LGM) period demonstrate how the scale of Mn limitation is sufficient to impact the response of Southern Ocean productivity and the carbon cycle. Ultimately, relief of Fe limitation in the Southern Ocean is compensated by an expansion of Mn limitation, an interplay that has likely driven the evolution of polar phytoplankton over millions of years.

## 2 Materials and Methods

### 2.1 Biogeochemical Cycles of Mn and Zn in the PISCES‐BYONIC Model

PISCES‐BYONIC is a global ocean biogeochemistry model based on PISCES‐v2 (Aumont et al., 2015), with the addition of global cycles of the micronutrients Mn, Zn, Cu, and Co (Hulten et al., 2017; Richon & Tagliabue, 2019, 2021; Tagliabue et al., 2018). The model simulates two phytoplankton functional types: a diatom‐like functional type that requires Si for growth, and a nanophytoplankton functional type. The two functional types differ in their nutrient uptake kinetics, assumed size and grazing loss (see Aumont et al. (2015) and Richon and Tagliabue (2021) for more details). The biological Fe cycle in PISCES‐v2 has also been modified to include regulation of Fe uptake rate under nitrogen‐limiting conditions for both functional types, consistent with recent measurements (Twining et al., 2021).

This study modified equations representing phytoplankton Mn uptake and added Mn growth requirements to the PISCES‐BYONIC model. Full details of the PISCES‐v2 model can be found in Aumont et al. (2015) and equations governing the Mn and Zn biogeochemical cycles are fully described in the Supporting Information of Richon and Tagliabue (Richon & Tagliabue, 2021). Briefly, the Mn model accounts for sources of Mn from atmospheric deposition, rivers, marine sediments, and hydrothermal vents. Mn in dust is assumed to be 25% soluble and the sediment supply is enhanced at both low oxygen and at higher organic carbon flux. In the model, dMn is removed by biological uptake and bacterially catalyzed precipitation of Mn oxides. Rates of bacterial scavenging of dMn increase with increasing temperature (Richon & Tagliabue, 2021; Tagliabue et al., 2018) but decrease when dMn falls below a threshold concentration, which is important for replicating the residual dMn inventory in the deep ocean (Hulten et al., 2017). High light and low oxygen also decrease the rate of Mn scavenging and enable the dissolution of Mn oxides, most notably in the illuminated surface mixed layer and in low oxygen water masses in the tropics, respectively. dMn is resupplied by zooplankton excretion, dissolution of Mn oxides and regeneration of sinking particulate organic material (Richon & Tagliabue, 2021).

The Zn cycle in the model accounts for external supply from rivers and dust, and internal cycling via biological uptake and regeneration, as well as reversible scavenging onto particulate organic carbon (Richon & Tagliabue, 2021; Weber et al., 2018). Bioavailable Zn is calculated by equilibrium with a single ligand at a fixed concentration of 1 nM. A small fraction of Zn uptake is also allocated to diatom frustules, and cycles in parallel to Si in the model (Weber et al., 2018). Modeled Mn and Zn cycles in PISCES‐BYONIC reproduce the major features of their oceanic distributions (Richon & Tagliabue, 2021; see also Figures S1 and S2 in Supporting Information S1).

### 2.2 The Minimum Mn Requirement

Minimum phytoplankton requirements for Mn are defined by a Manganese Use Efficiency (MnUE), whereby increasing Mn is needed to support increasing growth rates (Raven, 1990). The MnUE represents the rate that carbon biomass can be produced per catalytic Mn atom, having units of mol C day^−1^ (mol Mn)^−1^, and is described by:

(1) $${MnUE}_{i}=\frac{\mu_{i}}{Q_{Mn,Req,i}}$$

where *μ* is the specific growth rate (day^−1^) and *Q*_Mn,Req_ is the required quota, that is, the amount needed for photosynthesis and basal metabolism (units of mol Mn: mol C). The subscript *i* reflects separate calculations for diatom and nanophytoplankton functional types in the model. *Q*_Mn,Req,*i*_ is calculated as:

(2) $$Q_{Mn,Req,i}=Q_{Mn,\min}+4*\frac{Chl{:C}_{i}}{Chl:PSII}$$

Approximating growth of the cultured open ocean diatom *Thalassiosira oceanica* (Sunda, 1989; Sunda & Huntsman, 1983, 1986), *Q*_Mn,min_ is set to 1 μmol Mn:mol C at a reference growth rate of 1 day^−1^, equal to a MnUE of 10^6^ mol C (mol Mn)^−1^ day^−1^; see Table 1. Conceptually, this basal requirement accounts for Mn metalloenzymes such as Mn superoxide dismutase, arginase, carbonic anhydrase, among others (Jensen et al., 2019; McCain et al., 2021; Peers & Price, 2004; Twining & Baines, 2013). While it is likely that each of these Mn enzymes are uniquely regulated based on intracellular or extracellular conditions, the scope and extent of this regulation is poorly defined at present. We consider the constant non‐photosynthetic Mn requirement to be a relatively conservative approach that appears consistent with the observation of increasing Mn requirements with increasing growth rate described by Sunda and Huntsman (Sunda & Huntsman, 1998b), as well as more general theories of nutrient limitation (Droop, 1973; Raven, 1988).

**Table 1:** Model Parameters Added to the PISCES‐BYONIC Model for This Study

Parameter	Value	Units	Description	Reference
*Q*_Mn,min_	1.0	μmol mol^−1^	Mn requirement not associated with photosynthesis	(Sunda. (1989); Sunda & Huntsman. (1986, 1996, 1998b))
Chl:PSII	1,000	mol mol^−1^	Photosynthetic antennae size	Table S1 in Supporting Information S1
*K*_Mn, nano_	5 × 10^8^	M^−1^	Binding constant for Mn’ to Mn transporter	(Sunda & Huntsman. (2000))
*K*_Mn, diatom_	1.67 × 10^8^			
*K*_Zn,a_	5 × 10^8^	M^−1^	Binding constant for Zn’ to Mn transporter	(Sunda & Huntsman. (2000))
*K*_Zn,b nano_	1 × 10^9^	M^−1^	Binding constant for Zn’ to the high affinity Zn transporter	(Sunda & Huntsman. (2000))
*K*_Zn,b diatom_	0.33 × 10^9^			
*Q*’_Mn,max_	6	μmol mol^−1^	Maximum Mn quota	SXRF Observations (Figure 2)
*Q*’_Zn,max, nano_	30	μmol mol^−1^	Maximum Zn quota	SXRF Observations (Figure 2)
*Q*’_Zn,max, diatom_	40			

*Note.* The parameter values are assigned following laboratory and field observations where possible. Parameters describing Mn sources and sinks can be found in Richon and Tagliabue (2021).

The photosynthetic component of the Mn requirement is dictated by a dynamic chlorophyll scheme already simulated in PISCES‐v2 (Aumont et al., 2015), originally based on the photoacclimation model of Geider et al. (1997). The Mn quota associated with PSII is calculated from the variable Chl:C ratio by applying a fixed antennae size, represented as a Chlorophyll:PSII ratio (Chl:PSII), and a stoichiometry of 4 Mn atoms per PSII (Raven, 1990). The standard model uses a Chl:PSII ratio of 1,000 to simulate the global characteristics of diatom and nanophytoplankton functional types. This value is in the upper end of the range of both field and culture observations (Table S1 in Supporting Information S1), with the exception of recent characterizations of Southern Ocean phytoplankton (Strzepek et al., 2019). It should be noted that phytoplankton Fe limitation can lead to an uncoupling of the Chl antennae from the photosynthetic apparatus, giving rise to large apparent Chl:PSII ratios that do not represent functional antennae‐photosystem complexes (Behrenfeld & Milligan, 2013). This process is not included in the PISCES Chl parameterization scheme, and thus we chose the intermediate Chl:PSII ratio of 1,000 to reflect the functional antennae size in the model.

**Figure 1:**
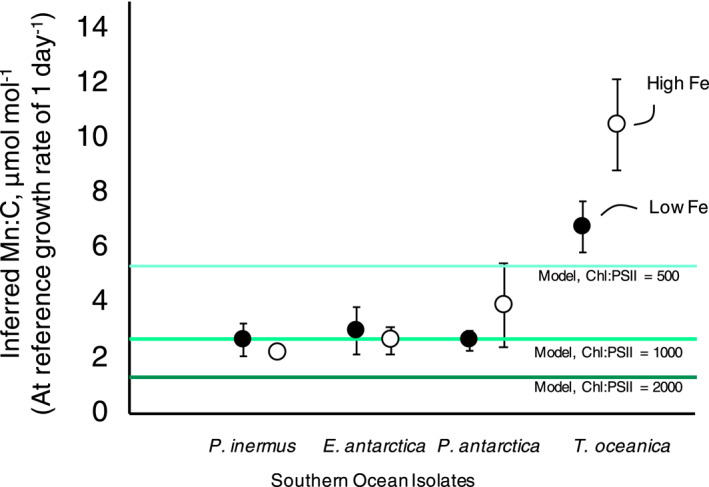
Estimated photosynthetic Mn:C requirements of three Southern Ocean phytoplankton isolates, the temperate diatom *T. oceanica*, and the diatom phytoplankton class in the PISCES‐BYONIC model. Estimates from culture experiments are based on the PSII Use Efficiency of Strzepek et al. (2019), which were conducted under low‐light conditions—where the photosynthetic apparatus is expected to be maximally upregulated—and under both high and low Fe availability (open and closed circles, respectively). The PSII Use Efficiency (units: mol C day^−1^ (mol PSII)^−1^) is converted to Mn:C by applying (i) a reference growth rate of 1 day^−1^, which normalizes across species‐specific maximum growth rates, and (ii) a Mn PSII^−1^ stoichiometry of 4:1. Model values (horizontal lines) reflect maximally upregulated Chl:C for the diatom class (0.05 g g^−1^) converted to Mn:C units with variable Chl:PSII ratios. This comparison suggests that the default Chl:PSII ratio of 1,000 in the PISCES‐BYONIC model is the best descriptor of these measurements, given the parametrization of Chl regulation, photosynthesis, and growth rates in the model (the higher reported Chl:PSII in Strzepek et al. (2019) of ∼2,000:1 leads to a much higher Chl:C ratio than is simulated in the phytoplankton functional types represented in this global biogeochemical model).

To ensure that this scheme was not overestimating Mn limitation for Southern Ocean phytoplankton, we calculated the Mn requirements inferred from the photo‐physiological data of Strzepek et al. (2019). PSII Use Efficiencies from that work (units: mol C (mol PSII)^−1^ day^−1^) were converted to Mn units via the Mn:PSII ratio (i.e., 4 mol Mn (mol PSII)^−1^; Figure 1). Averaging across the three Southern Ocean phytoplankton species characterized in Strzepek et al. (2019), grown at low irradiance under both low and high Fe availability, a relatively narrow range of photosynthetic Mn requirement is predicted: 2.85 ± 0.53 μmol Mn:mol C at a reference growth rate of 1 day^−1^ (note that, per Equation 1, this value decreases as growth rate decreases). We compared this to our model by applying the maximum Chl:C ratio of the diatom functional type (0.05 g Chl (g C)^−1^ or 673 μmol Chl:mol C, assuming a molar mass of 891 Da for chlorophyll *a* (Aumont et al., 2015)), the default antennae size of 1,000, and the Mn:PSII ratio of four. This calculation results in a photosynthetic Mn requirement equal to 2.69 μmol Mn: mol C (Figure 1), within the range of expected photosynthetic Mn requirements calculated from Southern Ocean diatoms (Strzepek et al., 2019). Applying the larger Chl:PSII ratio of 2,000 decreases this requirement to 1.35 μmol Mn: mol C, which appears to underestimate photosynthetic Mn requirements for these isolates (Figure 1).

In the model, the MnUE constrains phytoplankton growth rate, calculated as:

(3) $$\mu_{Mn,i}={MnUE}_{i}*Q_{Mn,i}$$

where *Q*_Mn_ is the realized Mn quota (in units of mol Mn: mol C, as per *Q*_Mn,Req_), set by dMn and other parameters influencing Mn uptake (see next section). Mn limitation of phytoplankton growth emerges when the Mn‐constrained growth rate falls below the background growth rate (i.e., *μ*_Mn_ < *μ*), the latter governed by light, temperature, Fe, N, P and Si (for diatoms). The overall strength of Mn‐limitation (dimensionless) is:

(4) $$\lim_{Mn,i}=\min\left(1,\frac{\mu_{Mn,i}}{\mu_{i}}\right)$$

While Mn‐deficiency is calculated as the ratio of *Q*_Mn_ to the maximum required Mn quota when other nutrients, most notably Fe in the Southern Ocean, are in excess. This term, *Q*_Mn,Req,max_, uses growth rate defined solely by temperature and light limitation (i.e., *μ*_replete,*i*_/MnUE_*i*_) and can fingerprint where Mn may emerge if Fe supply were to increase.

### 2.3 Phytoplankton Mn Uptake

Manganese uptake in both phytoplankton classes follows standard Michaelis‐Menten kinetics, modified to account for competitive inhibition due to Zn.

(5) $$\rho_{Mn,i}=V_{\max,Mn}*\left(\frac{K_{Mn}\left[Mn^{\prime }\right]}{K_{Zn,a}\left[Zn^{\prime }\right]+K_{Mn}\left[Mn^{\prime }\right]+1}\right)$$

where *K*_Mn_ represents the equilibrium binding affinity of the Mn transporter to Mn^2+^ and other inorganic Mn species in seawater, collectively termed Mn′. *K*_Zn,a_ represents the affinity of the same transporter to Zn′ (encompassing Zn^2+^ and other inorganic Zn species). The maximum uptake rate, *V*_max_, represents the number of transporter sites and a characteristic transport time. To reflect changes in the number of transporter sites, *V*_max_ is represented by the equation:

(6) $$V_{\max,Mn}=Q_{Mn\_max,i}*\mu_{\max,i}*R_{up,Mn}*R_{down,Mn}*R_{Zn}$$

where a maximum uptake rate, defined as the product of the maximum quota, *Q*_Mn___max,i_, and the maximum growth rate is modulated by three physiological regulation terms: (a) *R*_up_, transporter upregulation in response to low *Q*_Mn_, (b) *R*_down_, transporter downregulation in response to high *Q*_Mn_, and (c) *R*_Zn_, transporter downregulation in response to hyperaccumulation of internal Zn. Each of these behaviors have been observed in the open ocean diatom *T. oceanica* (Sunda & Huntsman, 1983, 1986, 2000). Corresponding experiments have not been performed for Antarctic species thus far, but polar lineages may have a greater ability to store and utilize intracellular Zn (Ye et al., 2022). As a result, *R*_Zn_ is set equal to one in the standard version of the model, which indicates no downregulation of Mn uptake in response to high *Q*_Zn_. It is important to note that this confers an adaptive advantage to the modeled diatoms beyond that observed in *T. oceanica* and coastal species (Sunda & Huntsman, 1996, 1998a; 1998b, 2000). For simplicity, we have not accounted for similar competitive inhibition by Cu’ and Cd’ because concentrations of these ions in the Southern Ocean (generally <10 pM) do not appear high enough to influence Mn uptake, in contrast to Zn^2+^ (Baars & Croot, 2011; Baars et al., 2014; Heller & Croot, 2015). We expect that this simplification may slightly underestimate the impact of Mn limitation in the Antarctic zone.

The maximum Mn cellular quota is adjusted from its prescribed value (Table 1) as a function of Mn requirements (*Q*_Mn,Req_) and the fractional nitrogen limitation term (lim_*N,i*_; range 0–1):

(7) $$Q_{Mn\_max,i}=Q_{Mn,Req}+\left(Q_{Mn\_max,i}^{\prime }-Q_{Mn,Req}\right)*\lim_{N,i}$$

**Figure 2:**
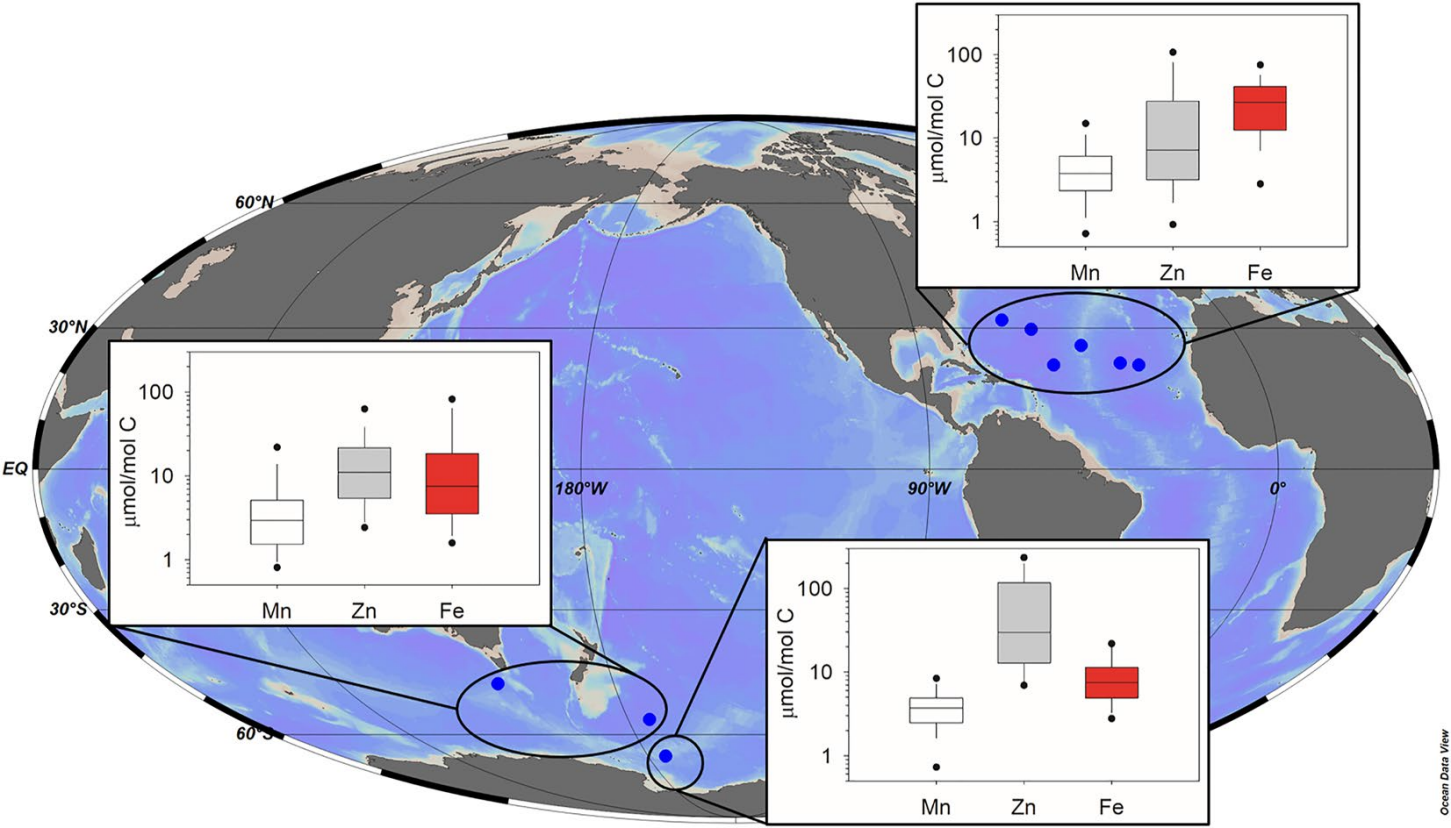
Comparison of phytoplankton Mn, Zn, and Fe quotas from three oceanic regions measured by single cell X‐ray fluorescence (SXRF). For SXRF measurements, horizontal lines represent the data set median value, box dimensions represent 25th and 75th quartiles, and whiskers cover the 10th and 90th percentiles. Black symbols indicate 5th and 95th percentiles. Data sources and number of cells analyzed are listed in Table S2 in Supporting Information S1.

This allows us to reconcile the relatively high *Q*_Mn_ observed under nutrient replete growth during culture experiments at high Mn′ with Single Cell X‐Ray Fluorescence (SXRF) measurements of Mn quotas, which remain relatively low in oligotrophic regions despite high dissolved Mn (Figure 2, Figure S3 in Supporting Information S1). An equivalent change to Fe and Zn maximum quotas is also implemented (Twining et al., 2021).

The upregulation function, *R*_up,Mn_, is defined as for Fe in the original PISCES‐v2 code:

(8) $$R_{up}=4-4.5\left(\frac{\min\left(1,\lim_{Mn}\right)}{0.5+\min\left(1,\lim_{Mn}\right)}\right)$$

and permits a maximum four‐fold upregulation when growth is strongly limited by Mn. The down‐regulation function, *R*_down,Mn_, also parallels that for Fe in PISCES‐v2:

(9) $$R_{down}=\max\left(0,\frac{1-f_{\max,Mn}}{1.05-f_{\max,Mn}}\right)$$

where:

(10) $$f_{\max,Mn}=\frac{Q_{Mn,i}}{Q_{Mn\_\max,i}}$$

So that the uptake rate decreases to zero as the ratio of *Q*_Mn_ to the maximum quota, *Q*_Mn_max_, approaches a value of one, avoiding build‐up of cellular Mn above *Q*_Mn_max_.

The Zn downregulation effect, *R*_down,Zn,_ is defined similarly to *R*_down,Mn_, following the equation:

(11) $$R_{Zn}=\max\left(0.1,\frac{1-f_{\max,Zn}}{1.05-f_{\max,Zn}}\right)$$

where:

(12) $$f_{\max,\mathrm{Zn}}=\frac{Q_{\mathrm{Zn},i}}{Q_{{\mathrm{Zn}}_{\max},i}}$$

except that the minimum downregulation by *R*_Zn_ is set to 0.1 (instead of 0 for *R*_down_). This avoids a complete shutdown of Mn uptake at high Zn′. Modeled Zn uptake is analogous to Mn, where uptake is proportional to Zn′ according to Michalis Menten kinetics:

(13) $$\rho_{Zn,i}=V_{\max,Zn}\left(\frac{K_{Zn,b}\left[Zn^{\prime }\right]}{K_{Zn,b}\left[Zn^{\prime }\right]+1}\right)$$

and

(14) $$V_{\max,\mathrm{Zn}}=Q_{\mathrm{Zn}\_\max,i}*\mu_{\max,i}*R_{\mathrm{up},\mathrm{Zn}}*R_{\mathrm{down},\mathrm{Zn}}$$

with *R*_up,Zn_ and *R*_down,Zn_ defined specifically for Zn in the same way as for Mn (Equations 8 and 9). In addition, *Q*_Zn_max,i_ is also decreased under *N* limitation as for Mn and Fe (Equation 7).

### 2.4 Model Experiments

The standard version of the PISCES‐BYONIC model presented here includes Mn growth limitation and accounts for transporter binding site competition between Mn and Zn via Equation 5. The standard model was integrated for 500 years using climatological offline physics fields to allow quasi equilibrium of the biogeochemical tracers. We then conducted a set of parallel sensitivity tests for 200 years, all initialized from the same initial state as the full model. The sensitivity tests were designed to examine how unique traits and possible adaptations of Southern Ocean phytoplankton could alter the impact of Mn limitation, and include: (a) a “no Mn limitation” run, in which phytoplankton growth in the full model was not affected by Mn, (b) a “no Zn interaction” run, where the transporter impact of Zn binding on Mn uptake was removed, (c) a “Mn transporter regulation by Zn hyperaccumulation” run, where down‐regulation of Zn uptake due to *Q*_Zn_ exceeding *Q*_Zn_max_ also down‐regulated Mn transport (i.e. Equation 11 was implemented), (d) a “very large photosynthetic antennae” run, where photosynthetic Mn requirements were derived assuming a Chl:PSII ratio of 2,000 mol mol^−1^ and (e) a “moderate photosynthetic antennae” run, where Mn costs were derived assuming a Chl:PSII ratio of 500 mol mol^−1^ (see Table S1 in Supporting Information S1). Finally, two additional model experiments were conducted where the standard model and the “no Mn limitation” model were forced by LGM dust inputs. These experiments utilized reconstructed LGM dust fluxes from Lambert et al. (2015) and increased the supply of both Fe and Mn relative to the standard model.

### 2.5 Single Cell X‐Ray Fluorescence (SXRF)

Carbon‐normalized Mn, Fe, and Zn quotas of individual cells in Figure 2 were compiled from published studies in the North Atlantic, Subantarctic, and Southern Oceans (Sofen et al., 2021; Twining et al., 2004, 2015). SXRF sample collection and analysis followed previously published methods using stringent trace metal clean techniques (Twining et al., 2011).

Individual cells from plankton populations are known to exhibit significant intra‐population variability (Bucci et al., 2012) and outliers were identified following Twining et al. (2019). Briefly, log‐transformed C‐normalized quotas were fit with an ANCOVA model (JMP, SAS) that included log (biovolume), station, and cell type (diatom or nanoflagellate) as effects. Individual quotas were removed from the data set if the Jackknife distances of the Studentized residuals of this model were greater than three; the probability of samples following outside this threshold is less than 1%, based on the *t* distribution. Approximately 1% of cell quotas in the data set were removed through this process. Additionally, Zn or Fe quotas >200 or >300 μmol (mol C)^−1^, respectively, were deemed to be impacted by abiotic material based on known physiological ranges (Sunda & Huntsman, 1995a, 1995b) and were removed. This affected less than five measurements in the data set.

## 3 Results and Discussion

### 3.1 Modeling Minimum and Realized Mn Quotas

**Figure 3:**
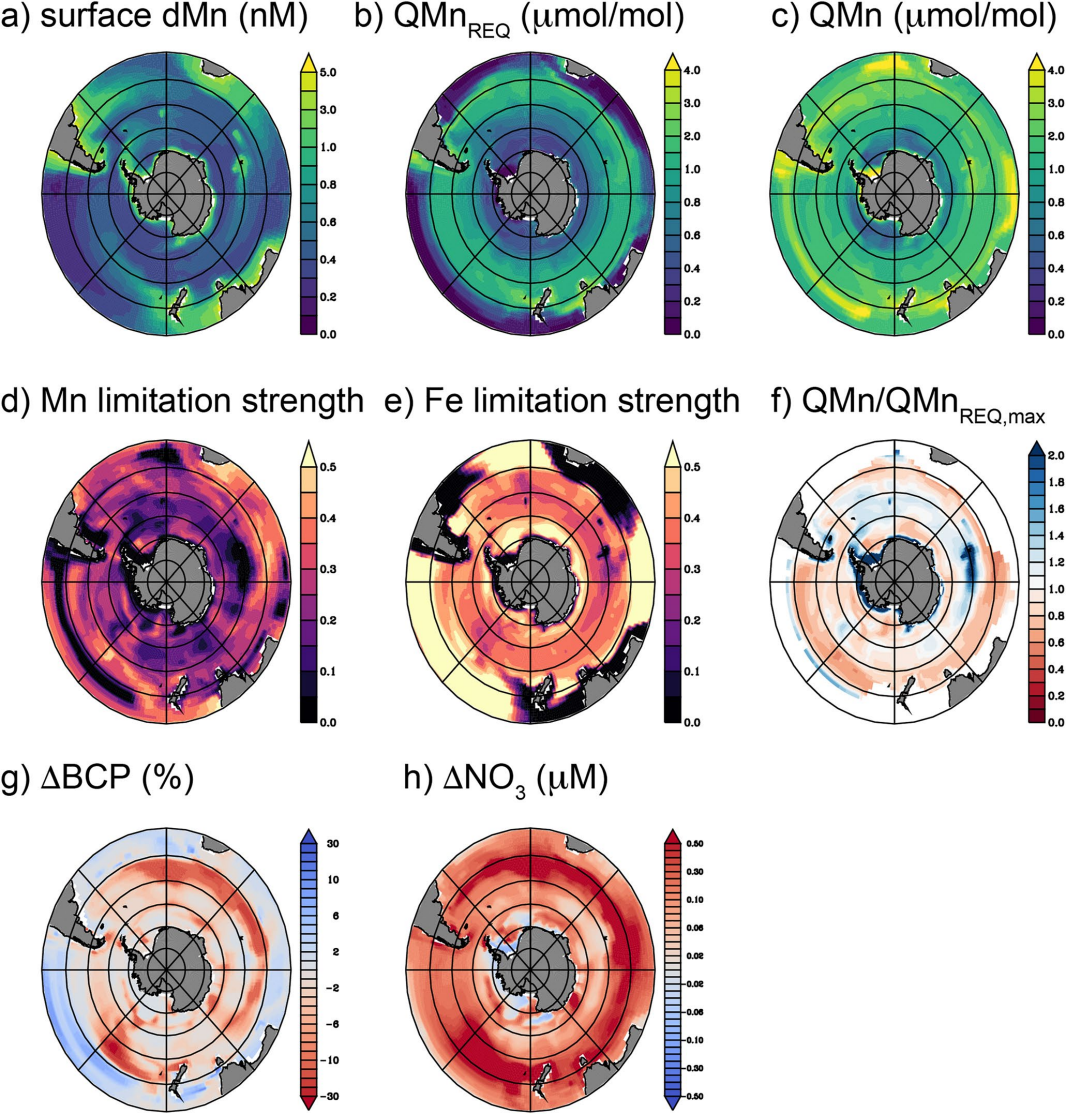
Southern Ocean Mn supply, demand, and limitation in PISCES‐BYONIC. Factors influencing Mn uptake and use by Southern Ocean phytoplankton in January at the surface (0–10 m), including (a) dissolved Mn, (b) the minimum Mn requirement, *Q*_Mn,REQ_ (μmol Mn/mol C), which is a function of phytoplankton chlorophyll and growth rate and (c) the Mn quota, *Q*_Mn_ (μmol Mn/mol C). (d) The seasonal maximum of the Mn limitation term, calculated as 1—lim_Mn_ (see Equation 4). Higher values indicate greater Mn limitation. (e) The equivalent Fe limitation term. (f) An index of Mn deficiency derived from the ratio of *Q*_Mn_ over *Q*_Mn,REQ,max_ (the Mn requirement associated with nutrient replete growth rates). Values below one indicate Mn is insufficient for replete growth. (g) The percentage change in the biological carbon pump, BCP (defined as the sinking particulate organic carbon flux across the 100 m depth horizon, integrated over the annual cycle) and (h) the change in standing stock of nitrate in the surface ocean (0–10 m) due to phytoplankton Mn limitation. Panels g and h reflect the difference between the standard model and a control model without Mn limitation terms and the red colors denote the impact of reduced productivity due to Mn limitation that diminishes the strength of the BCP and leaves increased residual nitrate stocks. Note that Panels a–c show January averages, while d–f show seasonal maxima, g is an annual integral and h is an annual maximum. In all Panels, concentric circles mark every 10° of latitude poleward of 30°S.

To quantify the impact of Mn scarcity on the Southern Ocean biological carbon pump, we incorporated phytoplankton Mn limitation into the PISCES‐BYONIC configuration of the global ocean biogeochemical model PISCES (Richon & Tagliabue, 2021). The PISCES‐BYONIC model represents limitation of phytoplankton growth by five nutrients: N, P, Si (for diatoms), Fe, and Mn, as well as light, and allows for variable cell chlorophyll and micronutrient quotas. The PISCES‐BYONIC model reproduces global patterns of dMn and dZn from GEOTRACES observations (Figure 3a, Figures S1 and S2 in Supporting Information S1), as well as dFe and other key biogeochemical properties (see Richon and Tagliabue (2021) and Tagliabue et al. (2016)).

Minimum cellular requirements for Mn (normalized to cell carbon: *Q*_Mn,Req_) are represented as the sum of the demand for oxygen‐evolving complexes in PSII and a basal requirement of Mn enzymes rooted in central metabolism. *Q*_Mn,Req_ also increases with increasing growth rate, as observed in both Fe fertilization experiments (Twining et al., 2004) and culture studies (Sunda & Huntsman, 1998b). Values of *Q*_Mn,Req_ are lowest in the subtropical gyres, where modeled growth rates are low, light is abundant and the photosynthetic apparatus is downregulated. Meanwhile, the stronger seasonality of irradiance and deeper mixed layers in the Southern Ocean cause greater Chl:C ratios that combine with episodes of relatively fast growth during Austral spring to drive maximum modeled *Q*_Mn,req_ in the Subantarctic zone between 40 and 50°S (Figure 3b).

Mn uptake in the PISCES‐BYONIC model is a function of bioavailable Mn’ and Zn’, which compete for the same transporter following experimental constraints (Sunda & Huntsman, 1996, 1998b). The combination of low dMn and high dZn of waters upwelling into the Southern Ocean leads to a minimum in *Q*_Mn_ between 60 and 70°S (Figure 3c). Close to the Antarctic continent and downstream of large islands (e.g., the Kerguelen Plateau region in the Indian Sector and the Weddell Sea), *Q*_Mn_ increases due to Mn inputs from margin sediments. However, Mn input to some coastal areas—notably the Ross Sea—appears insufficient to yield maximum *Q*_Mn_, which is consistent with recent reports of phytoplankton Mn‐Fe co‐limitation during summer (Wu et al., 2019).

### 3.2 Southern Ocean Footprints of Mn Limitation and Mn Deficiency

Based on modeled *Q*_Mn_ and *Q*_Mn,Req_, we calculated the proportion of maximum growth rate allowed by Mn (lim_Mn_; Equation 4) and subtracted this value from one to yield a unitless measure of “Mn limitation” (Figure 3d). Under this definition, higher values reflect more strongly Mn‐limiting conditions. While large areas of the Southern Ocean are predicted to be “Mn‐limited” to some extent, the model simulated a greater prevalence and intensity of Fe limitation in the same regions (Figure 3e), which is broadly consistent with literature compilations of nutrient amendment experiments in the Southern Ocean (Table S3 in Supporting Information S1). Although Fe limitation is much more prevalent in our model, local hotspots of Mn limitation reduce the strength of the biological carbon pump by 20% in all Southern Ocean sectors and by up to 30% in the Subantarctic Pacific (Figure 3g). For context, the decrease in the biological carbon pump by Mn limitation is similar in magnitude to the stimulating effect of hydrothermal Fe supply (Resing et al., 2015; Tagliabue & Resing, 2016). In PISCES‐BYONIC, the effects of Mn limitation are focused primarily in the Subantarctic between 40 and 50°S, especially in the Indian and Pacific sectors, with a smaller, patchier signal around 60°S (Figure 3g). Ultimately, the reduced efficiency of macronutrient utilization due to Mn limitation causes a small increase in the surface nitrate concentration (Figure 3h), which is redistributed from localized sites of Mn limitation throughout the Southern Ocean by lateral mixing. Unused Fe and macronutrients due to Mn limitation also fuels a small increase in carbon export in downstream subtropical waters (Figure 3g).

Beyond the small footprint for “proximal” Mn limitation, we found that *Q*_Mn_ over most of the Southern Ocean did not strongly exceed *Q*_Mn,Req_ (Figures 3b and 3c), indicating that Mn limitation might emerge rapidly with any increase in Fe supply. Indeed, the widespread Fe limitation typical of the Southern Ocean actually lowers *Q*_Mn,Req_ by enforcing slow growth rates. This indirect effect can be accounted for by defining a state of “Mn deficiency,” which instead normalizes *Q*_Mn_ to the amount of Mn required to support growth rates in the absence of Fe limitation (i.e., *Q*_Mn,Req,max_). Conceptually, our definition of Mn deficiency is similar to the additive responses observed in bottle incubations where simultaneous addition of both Mn and Fe increase biomass more than addition of Fe alone (Balaguer et al., 2022; Browning et al., 2021). In the standard version of the model, over half of the Southern Ocean experiences “Mn deficiency” at some point during the seasonal cycle (62% of waters poleward of 40°S; Figure 3f).

### 3.3 Seasonal Phasing of Mn Limitation

The seasonal dynamics of ocean mixing across the Southern Ocean decouple the supply of Mn and Fe, leading to seasonal evolution of nutrient limitation regimes. Winter mixing supplies Fe from the ocean interior and is the dominant input of Fe across the Southern Ocean, with the wintertime Fe stock then recycled by marine ecosystems throughout the spring and summer (Boyd et al., 2012; Strzepek et al., 2005; Tagliabue et al., 2014). In contrast to Fe, rates of Mn supply by winter mixing in the Southern Ocean are smaller (Rigby et al., 2020), as concentrations in the ocean interior are also low (Latour et al., 2021; Moore, 2016). In some regions, especially downstream of the Antarctic Peninsula, vertical mixing can still supply dMn from subsurface maxima that reflect Mn remineralization and recent interaction with continental shelves (Middag et al., 2011; Rigby et al., 2020).

**Figure 4:**
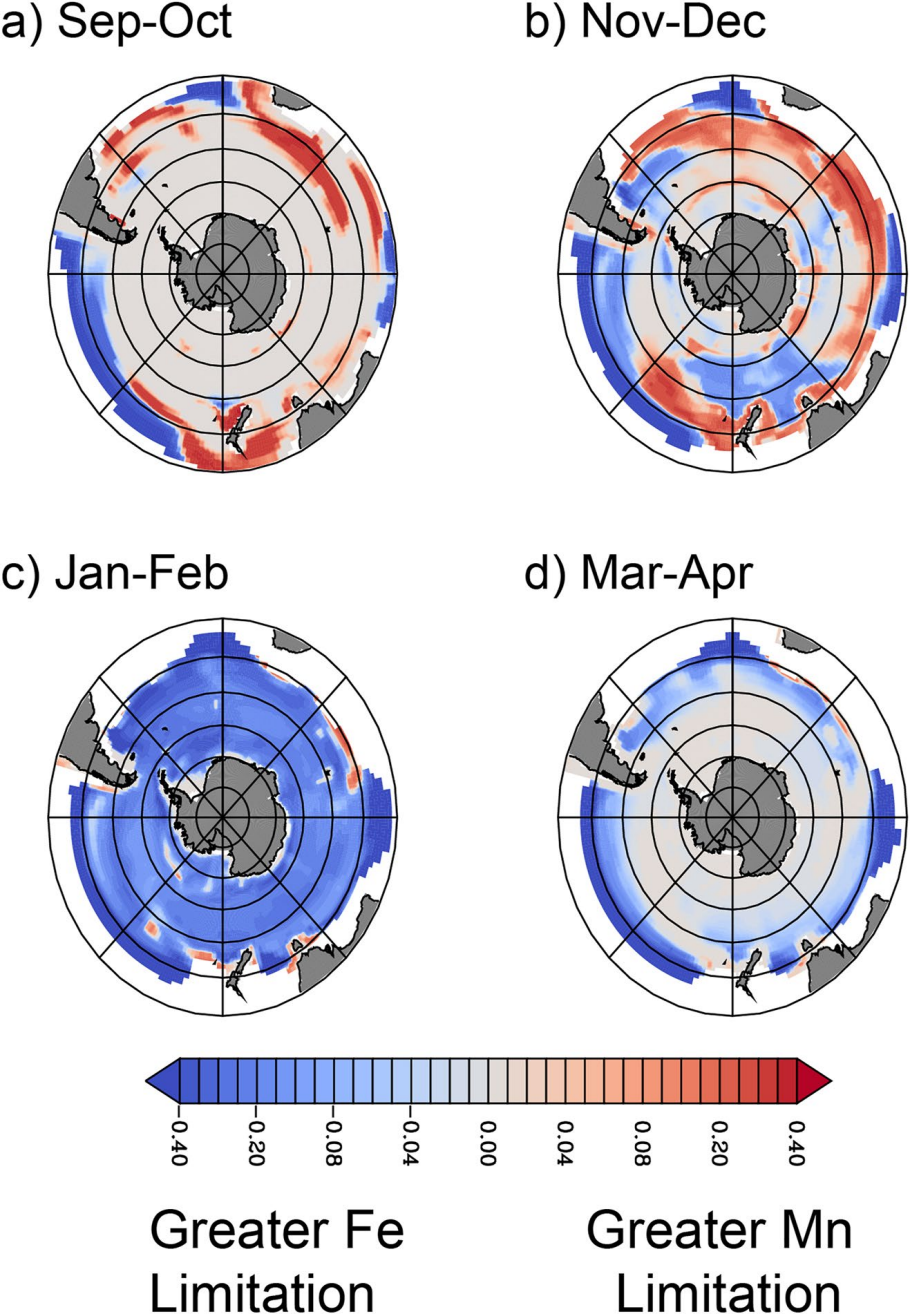
Seasonal phasing of Mn and Fe limitation. The difference between fractional Mn limitation and Fe limitation terms at the surface for (a) September–October, (b) November–December (c) January–February and (d) March–April, calculated as lim_Mn_—lim_Fe_ (see Equation 4). Positive values (in red) indicate greater Mn limitation than Fe limitation, while negative values indicate greater Fe limitation (blue). Nitrogen‐limited areas at low latitudes are masked in white. In all panels, concentric circles mark every 10° of latitude poleward of 30°S.

As the Southern Ocean stratifies during spring, a patchwork of localized Mn limitation emerges, primarily in November and December (Figures 4a and 4b), hindering the progress of the austral spring bloom. During this period, Mn is more limiting than Fe in our model for 49% of surface waters south of 40°S. These waters are characterized by low dFe concentrations, but the winter Fe supply that persists into spring still permits higher growth rates than Mn. Meanwhile, sub‐optimal light levels keep phytoplankton Chl:C elevated. If antennae size is already maximized (assumed in our model by a fixed Chl:PSII ratio), the combination of low light and relatively fast growth rates increases *Q*_Mn,Req_, leading to Mn limitation throughout the Subantarctic. By January and February, the modeled mixed layer dFe reservoir is depleted, resulting in Fe limitation exceeding Mn limitation across 96% of the Southern Ocean south of 40°S (Figure 4c). The ecosystem then returns to light limitation when mixed layers deepen in autumn (Figure 4d). This seasonal phasing between Mn and Fe limitation is not predicted from simpler models that do not account for variable Mn requirements (Browning et al., 2021), but springtime Mn limitation allows the mixed‐layer Fe stock to persist later into summer (Boyd et al., 2012; Strzepek et al., 2005). The seasonal progression from Mn to Fe limitation in the model is also consistent with the prevalence of Fe limitation from prior Fe and Mn addition experiments, which have mostly been conducted in austral summer (see Table S3 in Supporting Information S1).

### 3.4 Phytoplankton Physiology and the Emergence of Mn Limitation

**Figure 5:**
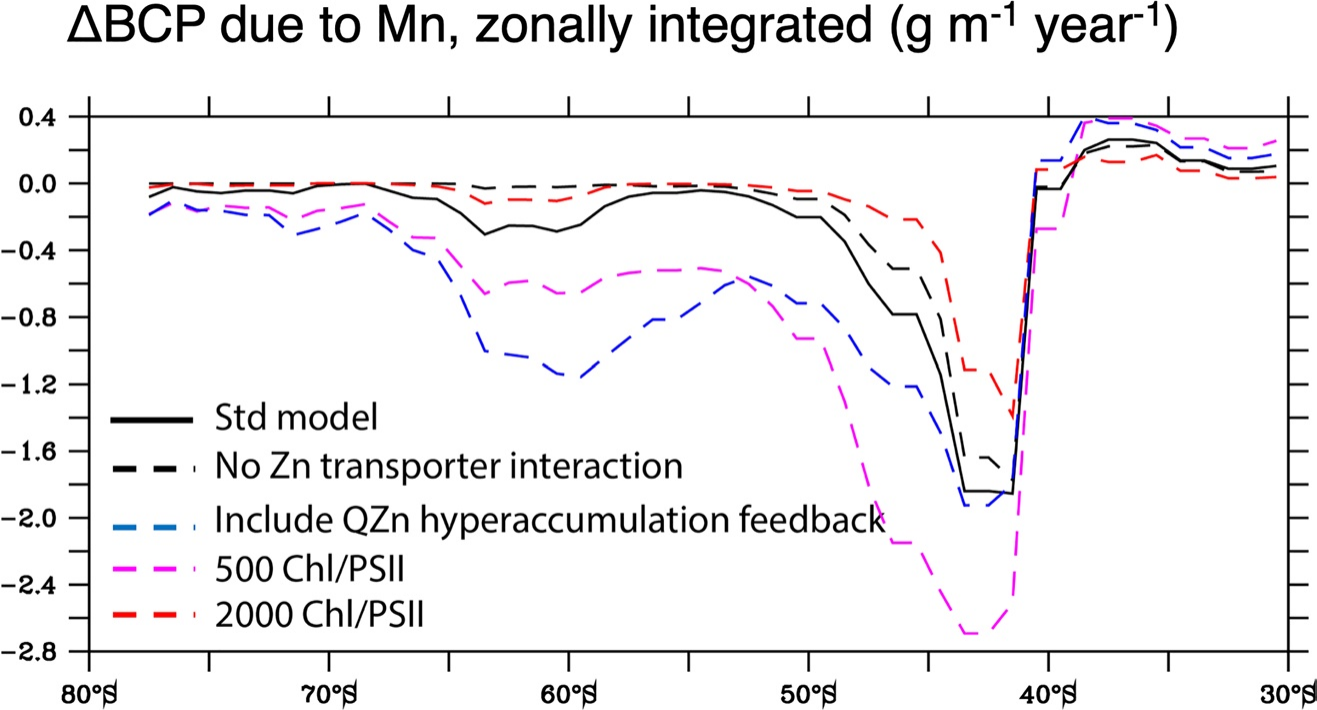
Influence of phytoplankton physiology on the severity of Mn limitation. The zonally and annually integrated anomaly in the biological carbon pump (ΔBCP) at 100 m depth (in grams carbon m^−1^ year^−1^) due to Mn limitation for the PISCES‐BYONIC standard model is shown (black line), along with four sensitivity experiments: a model without Zn‐Mn transporter competition (black dash), a model with Zn‐Mn transporter competition intact and an added downregulation of Mn transport to prevent Zn hyperaccumulation at high *Q*_Zn_ (blue dash), and the standard model with the upper (2,000:1) and lower (500:1) bounds of the assumed Chl:PSII ratio (red and purple dashes, respectively).

Because the physiological characterization of Southern Ocean phytoplankton is incomplete, we designed multiple sensitivity experiments to examine how specific traits related to Mn uptake and allocation could affect the severity of Mn limitation. When we removed Zn inhibition of Mn uptake, there was little change in the Subantarctic biological carbon pump anomaly caused by Mn limitation. However, the corresponding anomaly in the Antarctic Zone was eliminated because Mn uptake could now meet Mn requirements in these high Zn waters (Figure 5, Figure S4 in Supporting Information S1). Conversely, if the Zn‐Mn antagonism is exacerbated by enabling the downregulation of Mn transport at high *Q*_Zn_, a trait that has been observed in culture experiments (Sunda & Huntsman, 1996, 2000), then the impact of Mn limitation in the Antarctic zone is expanded greatly (Figure 5). This effect would be further increased if other divalent metals that compete for Mn transporters (Cu^2+^ and Cd^2+^) were also found to reach significant levels in the Antarctic zone. While traits related to Zn‐Mn interactions are central to the emergence of Mn limitation in the Antarctic Zone, the broad signal of Mn limitation throughout the Southern Ocean is regulated by photosynthetic traits. For instance, if the size of the photosynthetic antennae is increased to 2,000 Chl:1 PSII (Strzepek et al., 2019) or reduced to 500:1 (Kolber & Falkowski, 1993; Lawrenz et al., 2013), then the overall impact of Mn limitation is decreased or increased by nearly 50%, respectively (Figure 5).

### 3.5 Response of Mn Limitation to Changing Iron Supply During the Last Glacial Maximum

**Figure 6:**
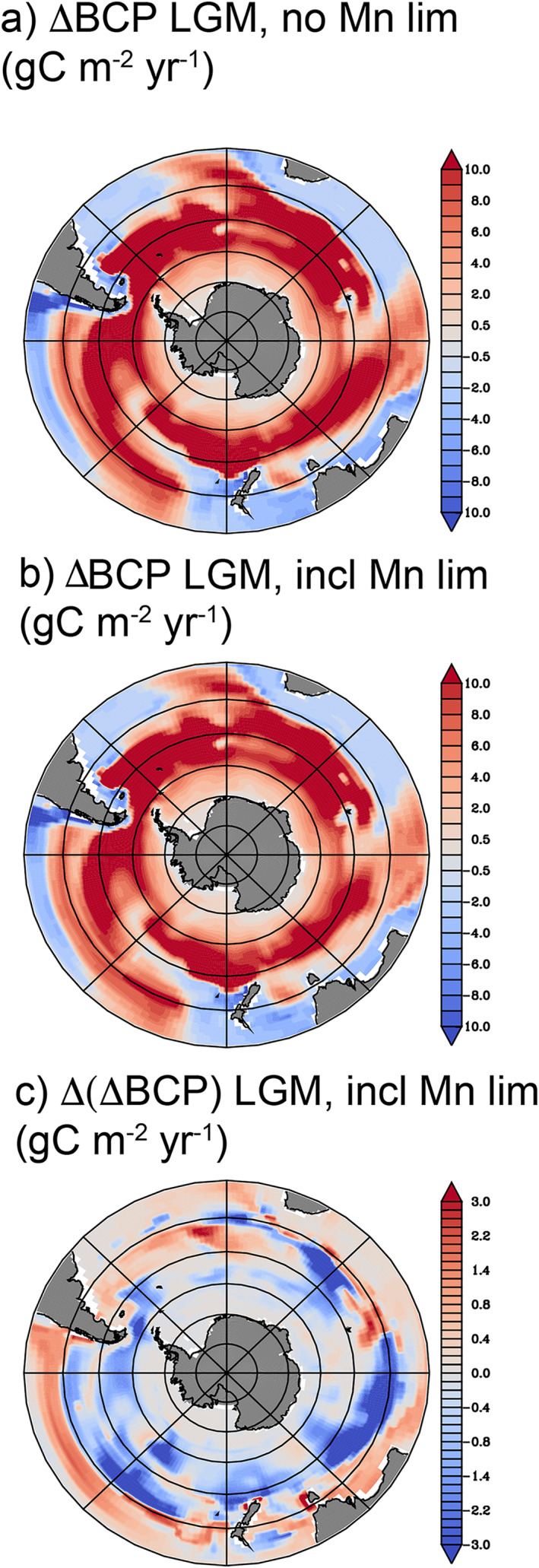
Expansion of Mn limitation with increasing dust supply. The change in the biological carbon pump (ΔBCP) in response to an increase in atmospheric dust supply of both Fe and Mn, based on projections for the Last Glacial Maximum (LGM). Simulation were performed for (a) a model without Mn limitation and (b) the standard PISCES‐BYONIC model with Mn limitation feedbacks. The difference (c) shows the impact of Mn on the ΔBCP responses to LGM dust, with blue shading indicating negative anomalies due to Mn limitation. Red areas in panel (c) are those where advection of residual nutrients stimulate the BCP in downstream subtropical regions, especially between 30 and 40°S. In all panels, concentric circles mark every 10° of latitude poleward of 30°S.

**Figure 7:**
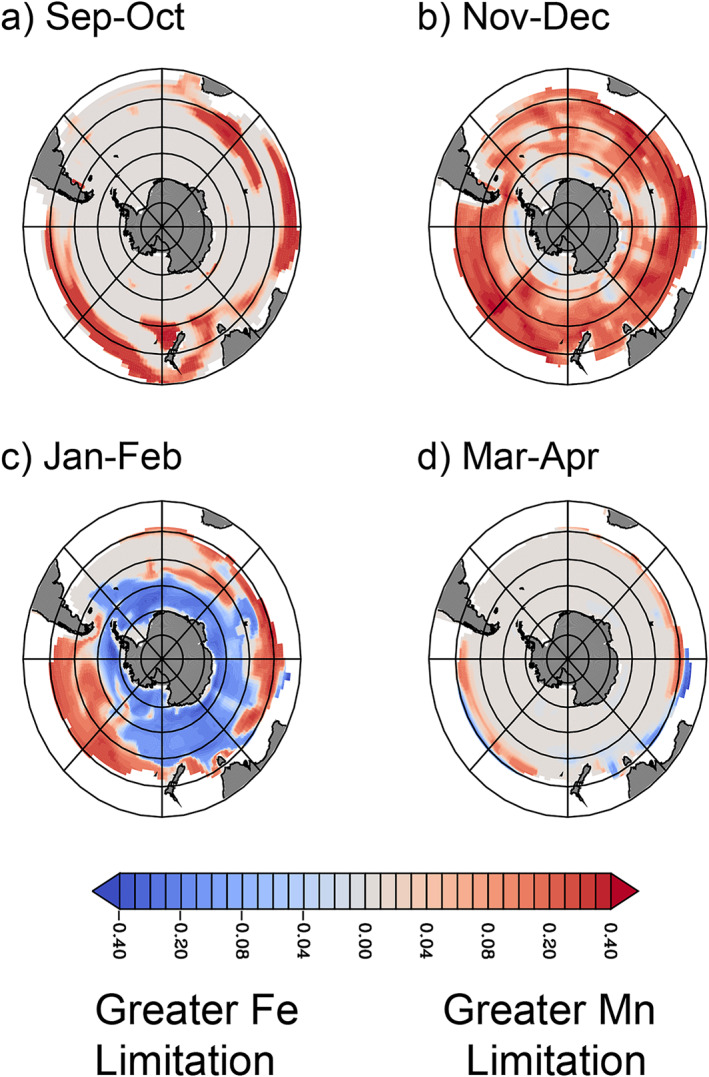
The difference between the fractional Mn limitation and Fe limitation term (normalized across both phytoplankton functional types in the model) for the Last Glacial Maximum (LGM) dust experiment at the ocean surface for (a) September–October, (b) November–December (c) January–February and (d) March–April. LGM dust deposition is elevated for all seasons. Color shading is identical to Figure 4. In all panels, concentric circles mark every 10° of latitude poleward of 30°S.

The prevalence of Mn deficiency throughout much of the Southern Ocean (Figure 3f) implies that there is the potential for Mn limitation to become more widespread when Fe supply increases. This is analogous to the LGM period, when atmospheric dust fluxes to the Southern Ocean were several‐fold greater than found today (Lamy et al., 2014; Martin, 1990). In an alternate version of our model without Mn limitation, the biological carbon pump is enhanced throughout the Southern Ocean when atmospheric dust supply is increased following paleo‐climate reconstructions of the LGM (Lambert et al., 2015), as expected (Figure 6a). However, when Mn limitation is considered (Figure 6b), the increase in the biological carbon pump is stunted by >30% across large regions of the Subantarctic Indian and Pacific sectors (Figure 6c). Even though the glacial dust scenario increases the supply of both Mn and Fe (with dust Mn much more soluble than Fe in our model; Chance et al., 2015; Richon & Tagliabue, 2021), the low abundance of Mn in dust means that the Mn:Fe ratio associated with dust deposition is below the range of phytoplankton Mn:Fe stoichiometry. As a result, Mn limitation exceeds Fe limitation throughout the Southern Ocean during spring, with Mn limitation in the Subantarctic Pacific and Indian sectors now persisting through summer (Figure 7). Although these simulations do not consider how parallel changes in ocean circulation may modulate phytoplankton growth conditions in the Southern Ocean, our mechanistic modeling results agree with a simpler diagnostic model (Browning et al., 2021). This provides new evidence that Mn was an important influence on the glacial carbon cycle across most of the Southern Ocean. Efforts to reconstruct glacial/interglacial changes in aeolian and sedimentary Mn sources, especially from the Antarctic continent, will be essential for refining these conclusions.

Earth System Models also predict enhanced Southern Ocean productivity by the end of the 21st century due to an increasing advective Fe supply from the subtropics and the warming and lengthening of the growth season due to sea ice melting (Misumi et al., 2014; Moore et al., 2018; Tagliabue et al., 2021). Our simulations suggest that any alleviation of Fe limitation will lead to an expanded impact of Mn deficiency that will add further hitherto unaccounted for uncertainty to future projections. Strzepek et al. (2019) hypothesized that large antennae may only be advantageous under cold temperatures, but experimental evidence showing a direct link between changing antennae sizes and temperature is currently lacking. If future work confirms that warmer temperatures do, in fact, select for smaller photosynthetic antennae sizes, our modeling indicates that this trait would increase phytoplankton Mn requirements and expand the impact of Mn limitation (Figure 5).

## 4 Wider Implications

### 4.1 Toward Improved Representation of Southern Ocean Ecosystems in Global Models

In constructing the PISCES‐BYONIC model, we have used the limited observational data set of phytoplankton cell quotas from the Southern Ocean to validate model predictions. Although the model cannot account for the diversity of coexisting phytoplankton species represented in cell‐specific SXRF measurements (Twining et al., 2004), simulated cell quotas for Mn, Fe, and Zn follow the same large scale trends found in SXRF datasets (Figure 2). For instance, modeled *Q*_Fe_ is several fold greater in the iron‐rich North Atlantic compared to the low dFe Southern Ocean (Figure S5 in Supporting Information S1), while observed and modeled *Q*_Zn_ shows the opposite trend: *Q*_Zn_ peaks in the Antarctic, tracking gradients in dZn (Figures S2 and S6 in Supporting Information S1). To an extent, this matches expectations from culture experiments where cell quotas largely reflect bioavailable metal concentrations (Hudson & Morel, 1993). In contrast, SXRF measurements of *Q*_Mn_ are relatively similar between the Southern Ocean and North Atlantic, generally falling between 2 and 5 μmol Mn: mol C (Figure 2), despite order‐of‐magnitude differences in dMn between the North Atlantic and the Southern Ocean (Hatta et al., 2015; Latour et al., 2021; Middag et al., 2011). In our model, stabilization of *Q*_Mn_ is achieved by down‐regulating Mn, Fe, and Zn uptake rates when metal quotas reach a prescribed maximum and, additionally, when phytoplankton are *N*‐limited. This scheme still allows modeled *Q*_Mn_ to reach lower values in some Southern Ocean regions than observed by SXRF (Figure S3 in Supporting Information S1). There is a need for broader observational datasets of phytoplankton cell quotas, especially in the Southern Ocean, to improve our understanding on the factors shaping Mn quotas in situ.

The principle uncertainty in our modeling originates from the lack of physiological data for phytoplankton species isolated from the Southern Ocean. Our model currently simulates two phytoplankton functional types (diatoms and nanophytoplankton), each representing a balance of the large number of physiological traits contained within a diverse global phytoplankton population. However, evidence is emerging of unique traits associated with the growth, photophysiology and Fe demands of Southern Ocean phytoplankton (Strzepek et al., 2012, 2019; Trimborn et al., 2019). Thus far, experiments with Antarctic phytoplankton have focused primarily on responses to Fe and light limitation, and do not include key information associated with Mn uptake and the regulation of Mn transporters (which are described in temperate species, notably *T. oceanica* (Sunda & Huntsman, 1986, 2000)). For instance, it is not clear if polar phytoplankton can further optimize their Mn uptake systems, or if improvement is prevented by fundamental constraints on the specificity of Mn^2+^ versus Zn^2+^ binding described by the Irving‐Williams series. Figure 5 demonstrates that the modeled footprint of Mn limitation is sensitive to assumptions regarding transporter regulation and photosynthetic antennae size, but our standard model applies a Chl:PSII ratio that is a relatively good fit with expected Mn requirements of Antarctic phytoplankton (Figure 1) and also assumes a greater ability to tolerate high Zn than is evident from culture experiments with *T. oceanica* (Sunda & Huntsman, 2000). Furthermore, we have not accounted for the likely upregulation of Mn superoxide dismutase under Fe limitation, whose quantitative importance to *Q*_Mn_ is not well defined by culture experiments. Although our model considers Mn limitation to be independent of any co‐occurring Fe deficiency (as in Liebig's Law of the Minimum), both protein allocation models (McCain et al., 2021) and culture experiments (Pausch et al., 2019) have suggested that the combined effects of Mn and Fe deficiency might depress growth rates further than singular Fe or Mn limitation. This suggests that our projections of Mn limitation may be conservative. To accurately predict the influence of changing climate on the Southern Ocean biological carbon pump, future modeling efforts should consider expanding the number of functional types so that the unique aspects of Southern Ocean phytoplankton can be considered directly. This will require more culture and field studies focused on Antarctic phytoplankton to identify and constrain physiological responses and trade‐offs to Mn scarcity, in particular.

### 4.2 A New Paradigm for Seasonal Resource Regulation and Adaption of Southern Ocean Phytoplankton

**Figure 8:**
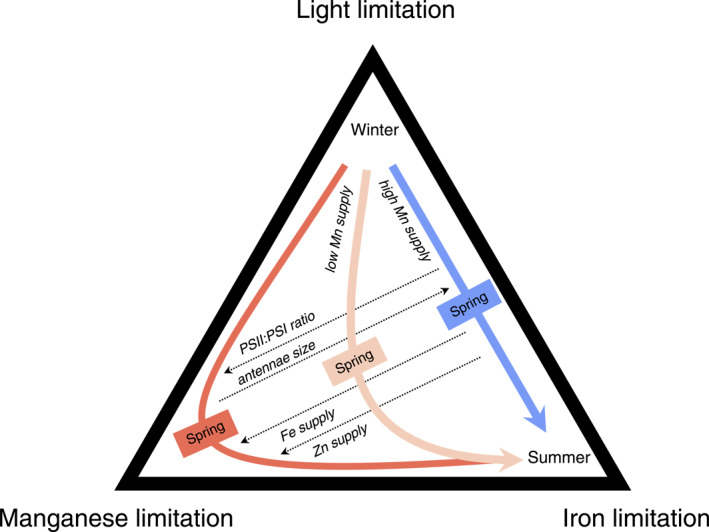
A new perspective on the seasonal transitions between iron, manganese, and light limitation in the Southern Ocean. Under conditions of high Mn supply, the light‐Fe limitation seasonal regime is dominant (blue arrow). As Mn supply decreases, a light‐Mn‐Fe limitation regime manifests (pink and red arrows). The intensity of the Mn‐limited component of the light‐Mn‐Fe limited regime is controlled by photosynthetic physiology (chlorophyll antennae size, PSII:PSI ratio) as well as the supply of Fe and Zn.

We find consistent emergence of phytoplankton Mn limitation under a range of potential physiological adaptations of polar phytoplankton. As a result, projections of past and future changes to Southern Ocean productivity should consider the impact of Mn, alongside the recognized roles of Fe and light. In regions of high Mn supply, the Southern Ocean biological pump oscillates seasonally between light limitation in winter and Fe limitation during summer. Elsewhere, the interaction between light, Mn and Fe will be an important component of the seasonal cycle (Figure 8). Proximal Mn limitation and co‐limitation manifests during periods of enhanced Fe availability and sub‐optimal light levels, particularly in the Subantarctic zone during spring and early summer. The extent of the light‐Mn‐Fe co‐limited regime over the year (Figure 8) depends on factors that regulate the demand for PSII (notably traits related to photosynthetic antennae size; Figure 5) or any other process that increases Mn requirements, such as elevated growth rates in response to Fe supply or increased expression of Mn superoxide dismutase and/or other Mn metalloenzymes under Fe limitation (McCain et al., 2021; Peers & Price, 2004). Neglecting to account for Mn limitation could mean that simulated responses of Southern Ocean ecosystems to climate change are incomplete.

The ultimate cause of Southern Ocean Mn and Fe deficiency is linked to the upwelling of deep ocean waters with high macronutrients and low Mn and Fe, conditions that have been in place since opening of the Drake Passage and the establishment of the Antarctic circumpolar current during the Eocene (Scher & Martin, 2006). As such, Southern Ocean phytoplankton have had millions of years to adapt to the simultaneous scarcity of Mn, Fe, and light, which may be reflected in their photosynthetic architecture. It is widely thought that selection for Fe‐conservation traits alone should lead to high PSII:PSI ratios in Southern Ocean phytoplankton, because Fe is mostly associated with PSI and alternate electron flows that avoid PSI are possible (Behrenfeld & Milligan, 2013; Strzepek & Harrison, 2004). Indeed, this strategy is borne out in temperate open‐ocean diatom lineages like *T. oceanica*, which show PSII:PSI exceeding 8:1, even when grown under very low irradiance (Strzepek & Harrison, 2004; Strzepek et al., 2019). In this context, the comparatively lower PSII:PSI ratio of ∼1.7 (range 1.3–2.0) observed in Fe‐limited Southern Ocean phytoplankton is enigmatic (Strzepek et al., 2019) because the presence of genes like plastoquinone terminal oxidase should also allow Antarctic phytoplankton to reach similarly high PSII:PSI ratios (Behrenfeld & Milligan, 2013; Moreno et al., 2018). It is possible that some, currently uncharacterized, polar phytoplankton may still employ this strategy. However, because all photosynthetic Mn is found in PSII, we estimate that increasing cellular PSII:PSI from 1.7 to 8 could triple photosynthetic Mn requirements relative to Fe (from 0.33 to 1.0 mol Mn: mol Fe; Figure S7 in Supporting Information S1). This would drastically increase the susceptibility to Mn limitation in the Mn‐deplete Southern Ocean. We posit that the comparatively low PSII:PSI ratios observed in polar phytoplankton may reflect an evolutionary trade‐off to optimize photosynthesis in a Southern Ocean that has long been deficient in both Fe and Mn.

## Supporting information

Supporting Information S1

## Data Availability

Model output from this work is publically available at https://doi.org/10.1029/2022GB007382.
