# Supplementary material for: Manganese Limitation of Phytoplankton Physiology and Productivity in the Southern Ocean
Source: Global Biogeochem Cycles. 2022 Nov 10;36(11):e2022GB007382. doi: 10.1029/2022GB007382 (PMC10078217; doi:10.1029/2022GB007382)
Supplement: Supplementary file 1 — Supporting Information S1 [file GBC-36-0-s001.pdf]

AGU PUBLICATIONS

Global Biogeochemical Cycles

Supporting Information for

Manganese Limitation of Phytoplankton Physiology and Productivity in the Southern Ocean

Nicholas J. Hawco[1]†, Alessandro Tagliabue[2]†, and Benjamin S. Twining[3]

[1] Department of Oceanography, University of Hawai‘i at Mānoa; Honolulu, HI, USA.

[2] School of Environmental Sciences, University of Liverpool; Liverpool, UK.

[3] Bigelow Laboratory for Ocean Sciences; East Boothbay Maine, USA.

Corresponding authors: Nicholas Hawco (hawco@hawaii.edu) and Alessandro Tagliabue: (a.tagliabue@liverpool.ac.uk)

† These authors contributed equally to this work

Contents of this file

Figures S1 to S7
Tables S1 to S3
Supplemental References

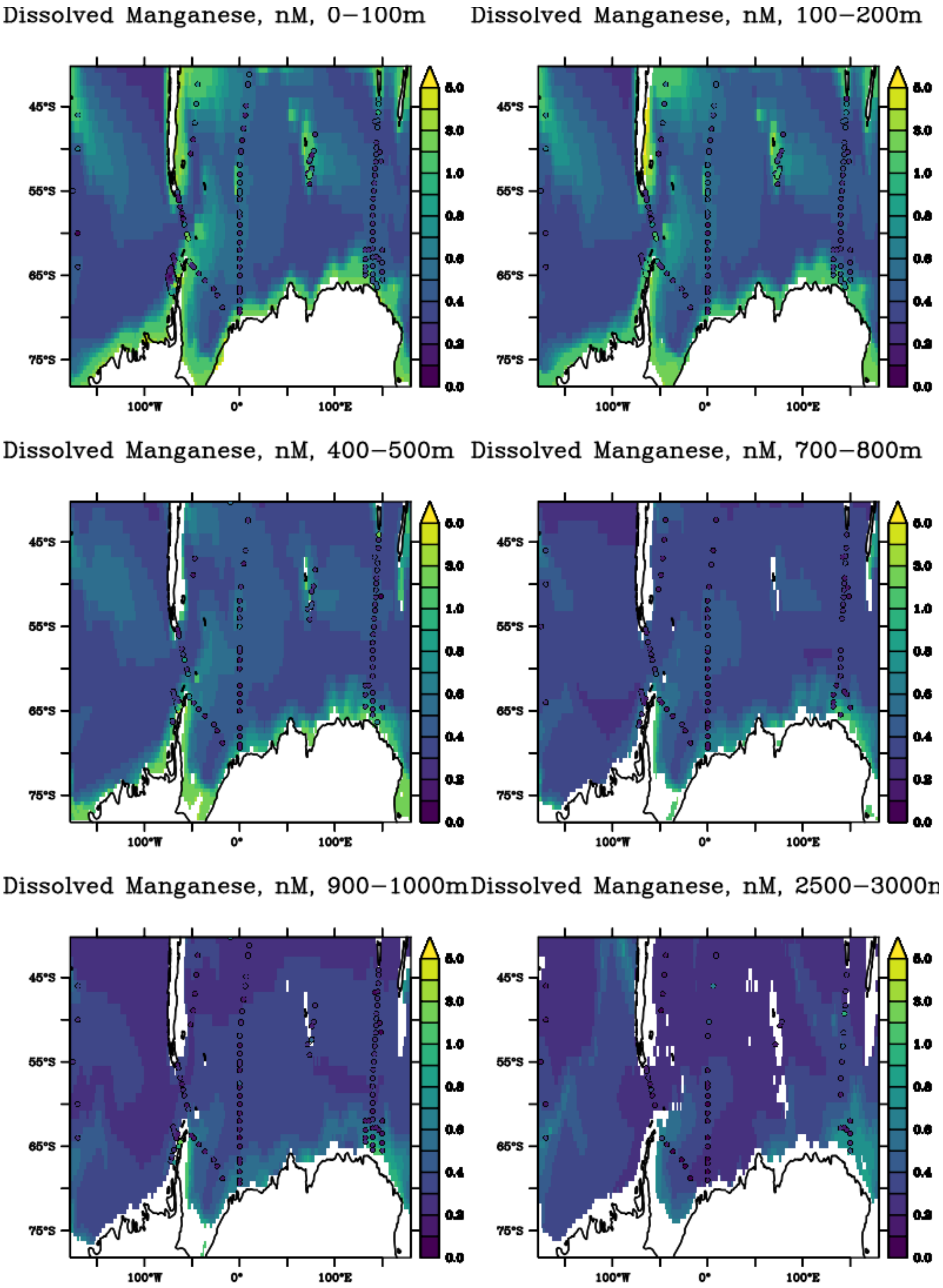

Fig. S1. Modelled dissolved manganese (dMn, nM, annual average) and compiled GEOTRACES observations from the Southern Ocean over various depth regions.

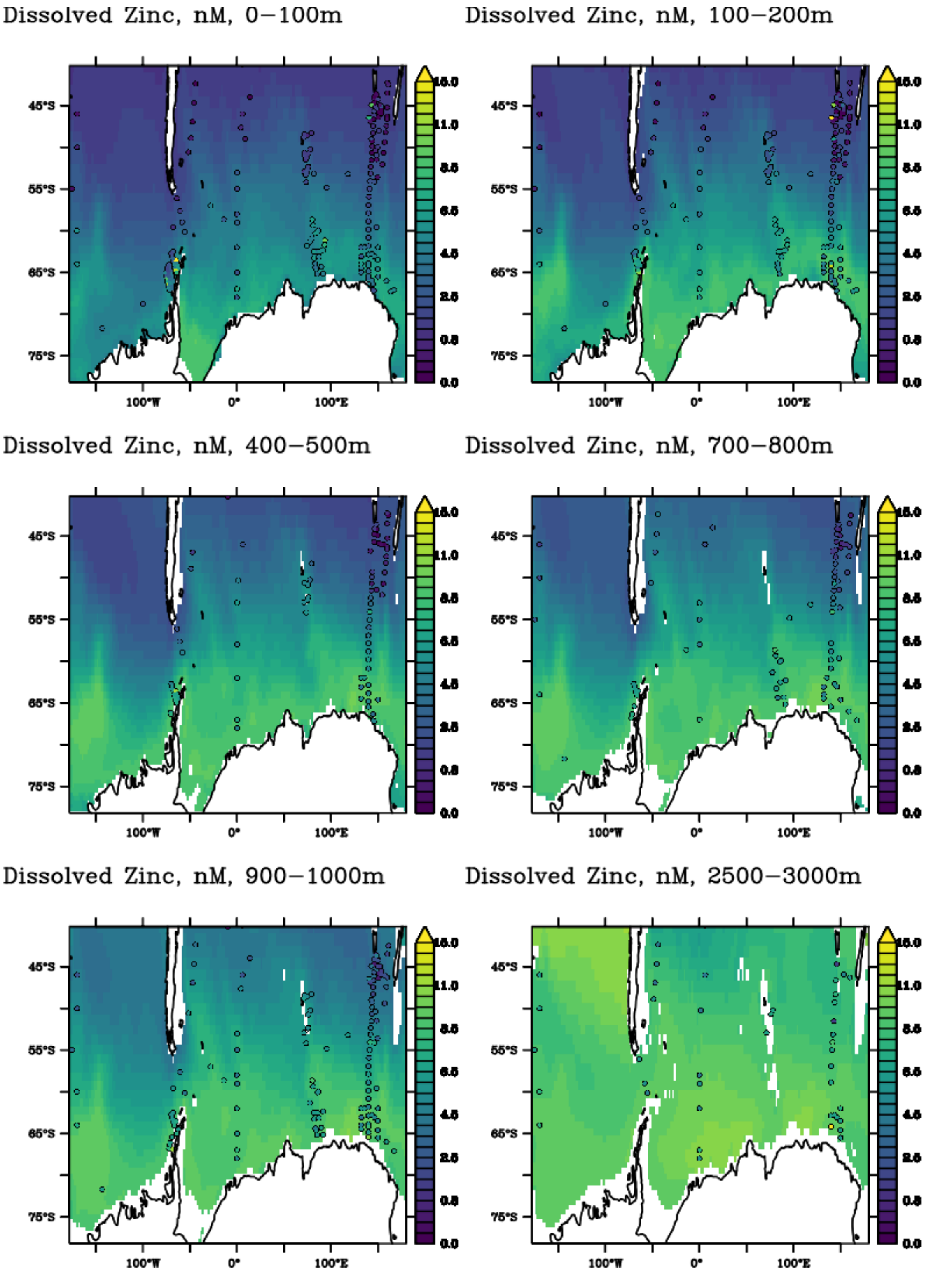

Fig. S2. Modelled dissolved zinc (dZn, nM, annual average) and compiled GEOTRACES observations from the Southern Ocean over various depth regions.

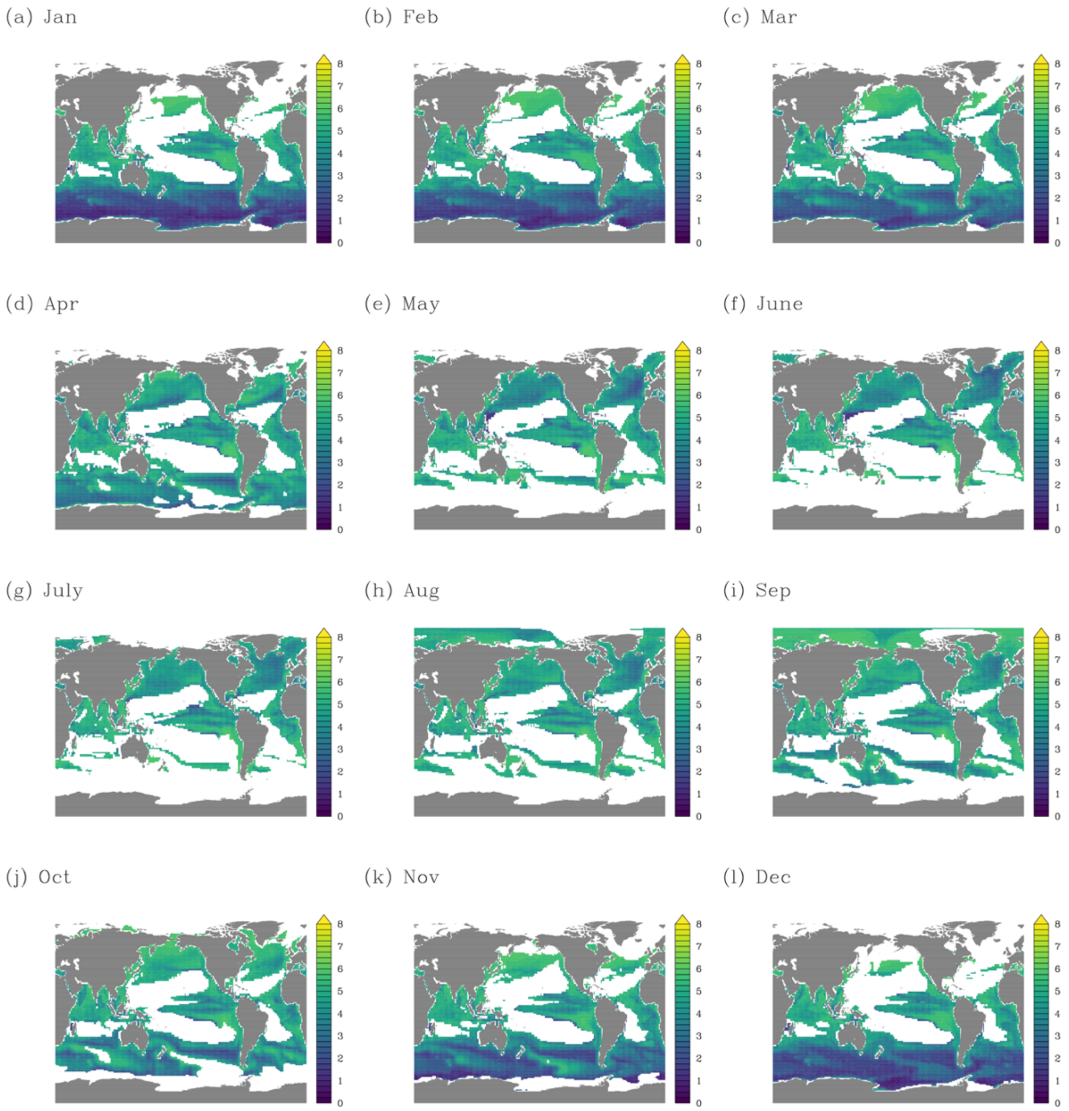

Fig. S3. Modelled surface (0-10m) Mn quotas across the global ocean in PISCES-BYONIC in in µmol Mn (mol C)$^{-1}$. Areas where phytoplankton carbon biomass falls below 1 x 10^{-6} mol C L^{-1} are masked in white.

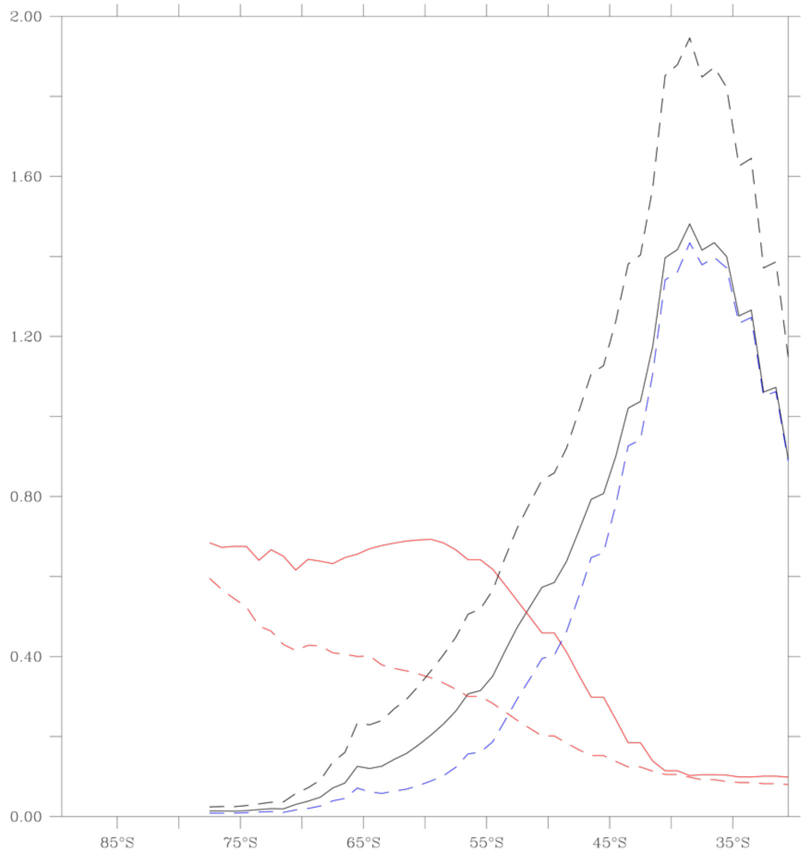

Fig. S4. Zonally integrated rates of Mn uptake (µmol m^{-1} $year^{-1}$) for the standard PISCES-BYONIC model (black line), and sensitivity experiments with 1) no Zn-Mn transporter competition (black dash), and 2) a feedback forcing downregulation of Mn transporters at elevated Q_{Zn} (blue dash). Also shown are zonal averages of dissolved Zn (in nM, divided by 10 for scale, red dash) and phytoplankton Zn quota normalized to the maximum Zn quota (range 0 – 1, red line). Compare with Figure 5 in the Main Text.

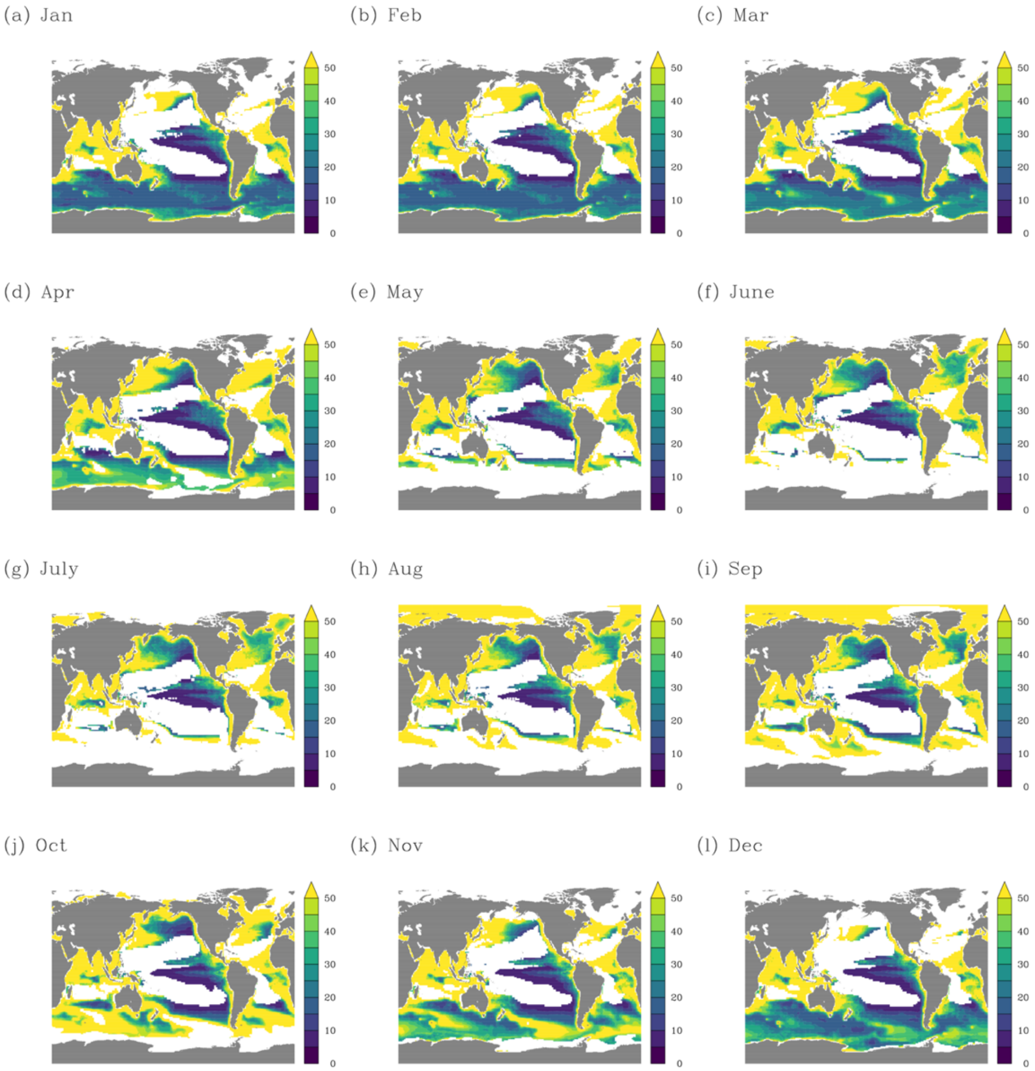

Fig. S5. Modelled surface (0-10m) Fe quotas across the global ocean in PISCES-BYONIC in µmol Fe (mol C)$^{-1}$. Areas where phytoplankton carbon biomass falls below 1 x 10^{-6} mol C L^{-1} are masked in white.

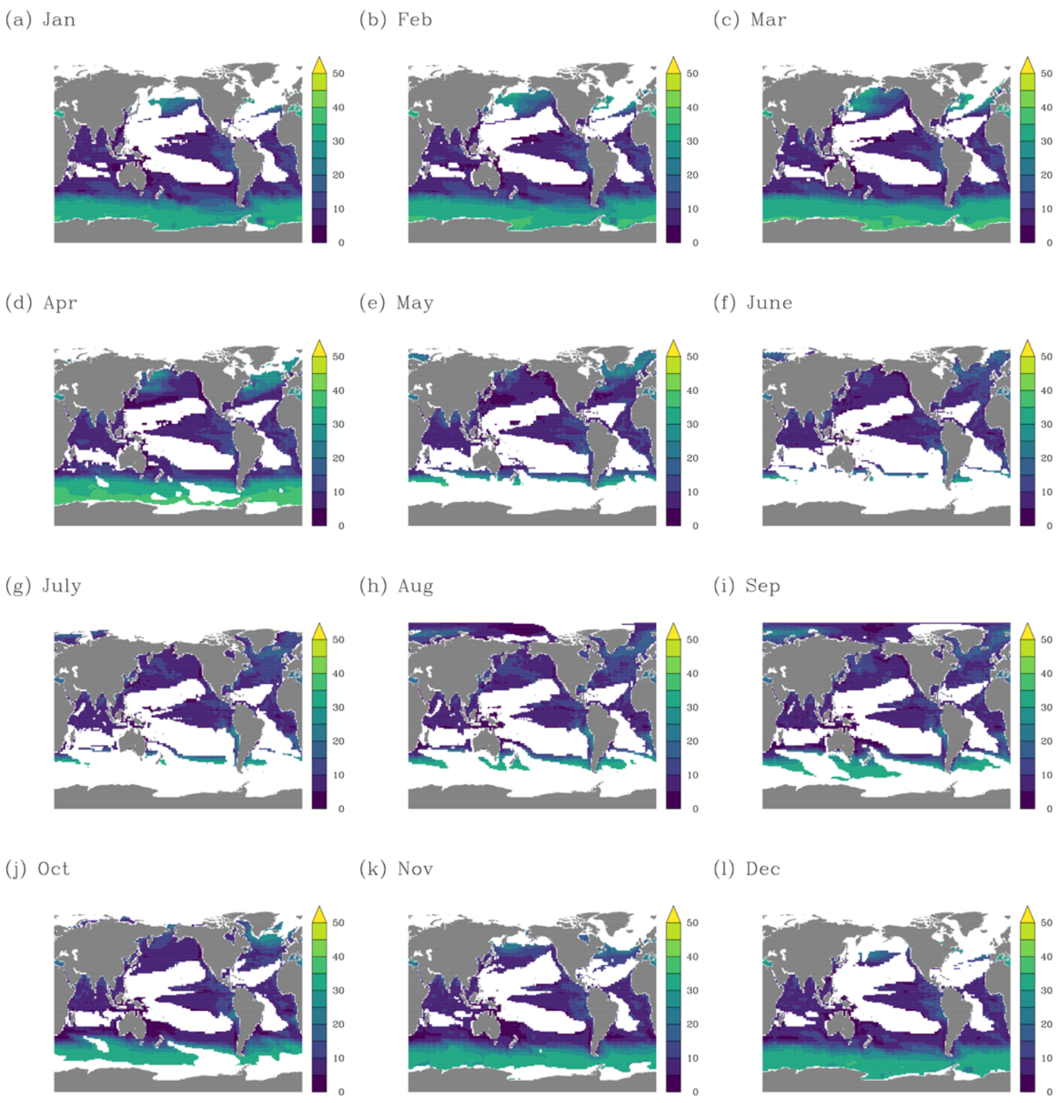

Fig. S6. Modelled surface (0-10m) Zn quotas across the global ocean in PISCES-BYONIC in µmol Zn (mol C)$^{-1}$. Areas where phytoplankton carbon biomass falls below 1 x 10^{-6} mol C L^{-1} are masked in white.

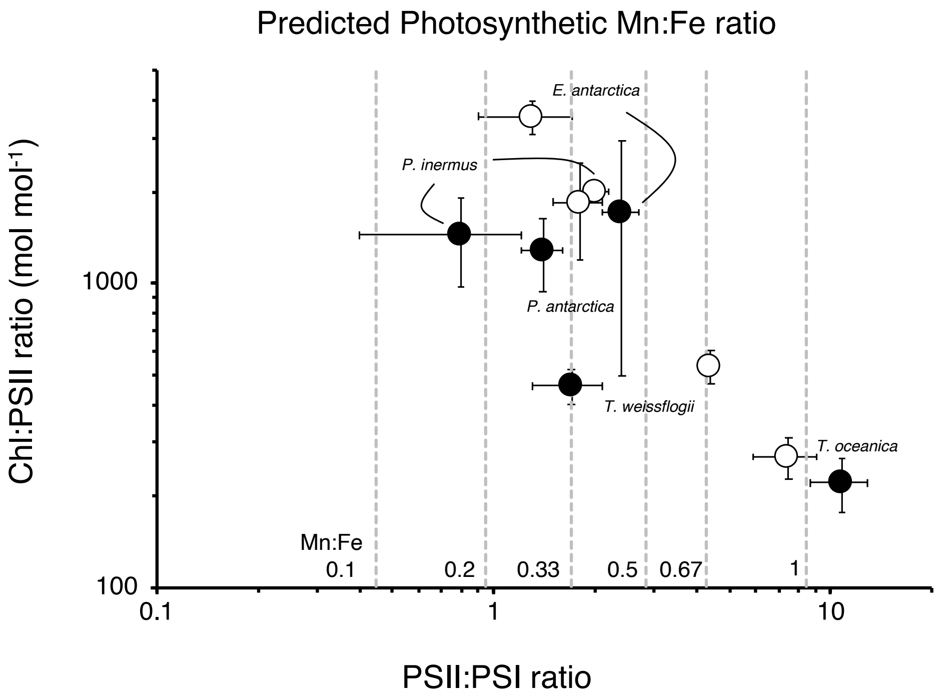

Figure S7. Comparison of PSII : PSI ratios and Chl : PSII ratios among Southern Ocean phytoplankton and the temperate diatoms *T. oceanica* and *T. weissflogii* using low iron (open circles) and high iron (filled circles) culture experiments from Strzepek et al. (2019). Dotted lines show predicted relative Mn : Fe requirements for photosynthesis (i.e. a ratio of 0.1 indicated 10-times more Fe is required than Mn). Required Mn:Fe ratios were calculated assuming 1) PSII contains 2 Fe atoms and 4 Mn atoms, 2) PSI contains 12 Fe and cytochrome b6 contains 5 Fe, and 3) that PSI and cytochrome b6 are in a 1:1 ratio. We note that *T. oceanica* is predicted to require as much Mn as Fe due to high PSII : PSI ratios, which is consistent with experimental data summarized in Sunda (1989).

Table S1. Estimated Chlorophyll *a*:PSII ratios from culture and field studies.

Organism / Region	Chl *a* : PSII (mol mol^{-1})	Reference
**Temperate Phytoplankton**		
Thalassiosira weissflogii	130 – 260	Dubinsky et al., 1986
	560 – 590	Suggett et al., 2004
	320 – 480	Strzepek & Harrison, 2004
	461 – 534	Strzepek et al., 2019
	1,000	Silsbe et al., 2015
Thalasiosira pseudonana	420 – 930	Sunda & Huntsman, 1998
	1,650	Silsbe et al., 2015
Thalassiosira oceanica	260 – 270	Strzepek & Harrison, 2004
	220 – 270	Strzepek et al., 2019
Skeletonema costatum	590 – 610	Falkowski et al., 1981
	1,151	Silsbe et al., 2015
Ditylum brightwellii	1,110	Silsbe et al., 2015
Phaeodactylum tricornutum	420 – 570	Friedman & Alberte, 1986
Chaetocerus muelleri	520 – 590	Suggett et al., 2004
	1,042	Silsbe et al., 2015
Dunaliella tertiolecta	590 – 620	Falkowski et al., 1981
	540 – 740	Suggett et al., 2004
Emiliana huxleyi	540 – 650	Suggett et al., 2004
	480 – 720	Suggett et al., 2007
	775	Silsbe et al., 2015
Isochrysis galbana	51 – 219	Dubinsky et al., 1986
Phaeocystis globosa	961	Silsbe et al., 2015
Aureococcus anophageffrens	720 – 950	Suggett et al., 2004
Prorocentrum minimum	260 – 365	Dubinsky et al., 1986
	430 – 530	Suggett et al., 2004
	725	Silsbe et al., 2015
Tetraselmis striata	790	Silsbe et al., 2015
Pycnococcus provasolii	621 – 930	Suggett et al., 2004
Rhodomonas salina	470 – 510	Suggett et al., 2004
Storeatula major	440 – 520	Suggett et al., 2004
Prochlorococcus strain SS120	270	Bibby et al., 2001, 2003
Synechococcus WH7803	240 – 290	Suggett et al., 2004
**Antarctic Phytoplankton**		
Phaeocystis antarctica	1280 – 1850	Strzepek et al., 2019
	630 – 1960	Trimborn et al., 2019
Proboscia inermis	1440 – 2070	Strzepek et al., 2019
Eucampia antarctica	1710 – 3540	Strzepek et al., 2019
Chaetoceros debilis	120 – 2540	Trimborn et al., 2019
**Field studies**		
Subtropical and Tropical Atlantic	330 – 420	Suggett et al., 2006
Celtic Sea	530 – 720	Moore et al., 2006
Subpolar North Atlantic*)*	380 – 1700	Macey et al., 2014
	400 – 833	Moore et al., 2005
Subarctic Pacific	280 – 450 (coastal)	Schuback & Tortell, 2019
	520 – 580 (open ocean)	
Southern Ocean	450 ± 350 (winter)	Ryan-Keogh et al., 2018
	1580 ± 1400 (summer)	
**Biogeochemical Model**		
Global	1000 (500 – 2000)	This study

Table S2. Summary of phytoplankton samples for Mn, Zn, and Fe quotas included in Figure 2. Number of analyzed cells from diatom and flagellate taxa (Flag) are listed.

Region	Cruise	Station	Lat (°N)	Lon (°E)	Depth (m)	Date	Diatom (n)	Flag (n)	Reference
Antarctic	SOFeX	19	-66	-172	20	24 Jan 2002	12	6	Twining et al., 2004
		27	-66	-172	20	2 Feb 2002	6	11	Twining et al., 2004
Subantarctic	SOFeX	7	-56	-172	20	12 Jan 2002	0	6	Twining et al., 2004
		11	-56	-172	20	20 Jan 2002	0	9	Twining et al., 2004
	SOTS	TM02	-47	142	15-30	7 Mar 2018	0	25	Sofen et al., 2021
		TM04	-47	142	15-40	9 Mar 2018	0	18	Sofen et al., 2021
		TM05	-47	142	15-30	18 Mar 2018	4	12	Sofen et al., 2021
N. Atlantic	GA02	2011-10	32	-64	25	19 Nov 2011	3	27	Twining et al., 2015
		2011-12	30	-57	25	23 Nov 2011	0	13	Twining et al., 2015
		2011-16	26	-45	25	30 Nov 2011	2	22	Twining et al., 2015
		2011-20	22	-36	25	3 Dec 2011	0	9	Twining et al., 2015
	ZIPLoC	2	22	-54	40	11 July 2017	0	22	Sofen et al., 2021
		7	22	-31	40	5 Aug 2017	0	15	Sofen et al., 2021

Table S3. Summary of published Southern Ocean Mn addition bio-assays.

Study / Reference	Region	Lat (°N)	Lon (°E)	Month	Limiting Nutrient
Balaguer et al., 2022	Drake Passage (West)	-60.4	-66.3	Mar	Fe
	Drake Passage (East)	-58.9	-60.9	Mar	Fe/Mn
Browning et al., 2021	Drake passage (Northern)	-54.7	-58.0	Nov	Fe
		-55.4	-57.7	Nov	Fe
		-55.6	-58.0	Nov	Fe
		-55.8	-57.8	Nov	Fe
	Drake Passage (Central)	-56.6	-57.4	Nov	Mn
		-56.8	-57.2	Nov	Mn/Fe
		-58.1	-56.4	Nov	Mn
	Drake Passage (Southern)	-58.7	-56.1	Nov	Fe
		-59.6	-55.5	Nov	Fe
		-61.0	-54.6	Nov	Replete
Wu et al., 2019	Ross Sea (McMurdo Sound)	-77.62	165.4	Dec	Replete
		-77.62	165.4	Jan	Mn/Fe
Sedwick et al., 2000	Ross Sea	-76.3	-179.6	Nov	Replete
		-76.3	-177.5	Dec	Replete
		-75	-172	Dec	Fe
		-76.3	-117.4	Jan	Fe
Sedwick & DiTullio, 1997	Ross Sea	-76.3	-170.4	Dec	Fe
		-76.3	-170.4	Jan	Fe
Scharek et al., 1997	Atlantic Sector, Polar Front	-47	-6	Oct/Nov	Fe
		-50	-6	Oct/Nov	Fe
	Atlantic Sector, ACC	-53	-6	Oct/Nov	Fe
	Weddell Sea	-59	-6.2	Oct/Nov	Fe
Buma et al., 1991	Weddell/ACC confluence (EPOS 158)	-59	-49	Dec	Mn/Fe
	Weddell Sea	-62	-47	Dec	Fe
	Scotia Sea	-57	-49	Dec	Fe
Martin et al., 1990	Ross Sea	-75	-173	Jan/Feb	Fe
		-72	167	Jan/Feb	Fe

Supplemental References

Balaguer, J., Koch, F., Hassler, C., & Trimborn, S. (2022). Iron and manganese co-limit the growth of two phytoplankton groups dominant at two locations of the Drake Passage. *Communications Biology*, *5*(1), 1–12.

Bibby, T. S., Nield, J., Partensky, F., & Barber, J. (2001). Antenna ring around photosystem I. *Nature*, *413*(6856), 590.

Bibby, T. S., Mary, I., Nield, J., Partensky, F., & Barber, J. (2003). Low-light-adapted Prochlorococcus species possess specific antennae for each photosystem. *Nature*, *424*(6952), 1051–1054.

Browning, T. J., Achterberg, E. P., Engel, A., & Mawji, E. (2021). Manganese co-limitation of phytoplankton growth and major nutrient drawdown in the Southern Ocean. *Nature Communications*, *12*(1), 884. https://doi.org/10.1038/s41467-021-21122-6

Buma, A. G. J., De Baar, H. J. W., Nolting, R. F., & Van Bennekom, A. J. (1991). Metal enrichment experiments in the Weddell-Scotia Seas: Effects of iron and manganese on various plankton communities. *Limnology and Oceanography*, *36*(8), 1865–1878.

Dubinsky, Z., Falkowski, P. G., & Wyman, K. (1986). Light harvesting and utilization by phytoplankton. *Plant and Cell Physiology*, *27*(7), 1335–1349.

Falkowski, P. G., Owens, T. G., Ley, A. C., & Mauzerall, D. C. (1981). Effects of growth irradiance levels on the ratio of reaction centers in two species of marine phytoplankton. *Plant Physiology*, *68*(4), 969–973.

Friedman, A. L., & Alberte, R. S. (1986). Biogenesis and light regulation of the major light harvesting chlorophyll-protein of diatoms. *Plant Physiology*, *80*(1), 43–51.

Macey, A. I., Ryan-Keogh, T., Richier, S., Moore, C. M., & Bibby, T. S. (2014). Photosynthetic protein stoichiometry and photophysiology in the high latitude North Atlantic. *Limnology and Oceanography*, *59*(6), 1853–1864.

Martin, J. H., Fitzwater, S. E., & Gordon, R. M. (1990). Iron deficiency limits phytoplankton growth in Antarctic waters. *Global Biogeochemical Cycles*, *4*(1), 5–12.

Moore, C. M., Lucas, M. I., Sanders, R., & Davidson, R. (2005). Basin-scale variability of phytoplankton bio-optical characteristics in relation to bloom state and community structure in the Northeast Atlantic. *Deep Sea Research Part I: Oceanographic Research Papers*, *52*(3), 401–419.

Moore, C. M., Suggett, D. J., Hickman, A. E., Kim, Y.-N., Tweddle, J. F., Sharples, J., et al. (2006). Phytoplankton photoacclimation and photoadaptation in response to environmental gradients in a shelf sea. *Limnology and Oceanography*, *51*(2), 936–949.

Ryan-Keogh, T. J., Thomalla, S. J., Little, H., & Melanson, J. (2018). Seasonal regulation of the coupling between photosynthetic electron transport and carbon fixation in the Southern Ocean. *Limnology and Oceanography*, *63*(5), 1856–1876.

Scharek, R., Van Leeuwe, M. A., & De Baar, H. J. W. (1997). Responses of Southern Ocean phytoplankton to the addition of trace metals. *Deep Sea Research Part II: Topical Studies in Oceanography*, *44*(1–2), 209–227.

Schuback, N., & Tortell, P. D. (2019). Diurnal regulation of photosynthetic light absorption, electron transport and carbon fixation in two contrasting oceanic

environments. *Biogeosciences*, *16*(7), 1381–1399.
Sedwick, P. N., & DiTullio, G. R. (1997). Regulation of algal blooms in Antarctic shelf waters by the release of iron from melting sea ice. *Geophysical Research Letters*, *24*(20), 2515–2518.
Sedwick, P. N., DiTullio, G. R., & Mackey, D. J. (2000). Iron and manganese in the Ross Sea, Antarctica: Seasonal iron limitation in Antarctic shelf waters. *Journal of Geophysical Research: Oceans*, *105*(C5), 11321–11336.
Silsbe, G. M., Oxborough, K., Suggett, D. J., Forster, R. M., Ihnken, S., Komárek, O., et al. (2015). Toward autonomous measurements of photosynthetic electron transport rates: An evaluation of active fluorescence-based measurements of photochemistry. *Limnology and Oceanography: Methods*, *13*(3), 138–155.
Sofen, L. E., Antipova, O. A., Ellwood, M. J., Gilbert, N. E., LeCleir, G. R., Lohan, M. C., et al. (2021). Trace metal contents of autotrophic flagellates from contrasting open-ocean ecosystems. *Limnology and Oceanography Letters*.
Strzepek, R. F., & Harrison, P. J. (2004). Photosynthetic architecture differs in coastal and oceanic diatoms. *Nature*, *431*(7009), 689–692.
Strzepek, R. F., Boyd, P. W., & Sunda, W. G. (2019). Photosynthetic adaptation to low iron, light, and temperature in Southern Ocean phytoplankton. *Proceedings of the National Academy of Sciences*, *116*(10), 4388–4393.
Suggett, D. J., MacIntyre, H. L., & Geider, R. J. (2004). Evaluation of biophysical and optical determinations of light absorption by photosystem II in phytoplankton. *Limnology and Oceanography: Methods*, *2*(10), 316–332.
Suggett, D. J., Moore, C. M., Marañón, E., Omachi, C., Varela, R. A., Aiken, J., & Holligan, P. M. (2006). Photosynthetic electron turnover in the tropical and subtropical Atlantic Ocean. *Deep Sea Research Part II: Topical Studies in Oceanography*, *53*(14–16), 1573–1592.
Suggett, D. J., Le Floc'H, E., Harris, G. N., Leonardos, N., & Geider, R. J. (2007). Different strategies of photoacclimation by two strains of Emiliania huxleyi (Haptophyta) 1. *Journal of Phycology*, *43*(6), 1209–1222.
Sunda, W. G. (1989). Trace metal interactions with marine phytoplankton. *Biological Oceanography*, *6*(5–6), 411–442.
Sunda, W. G., & Huntsman, S. A. (1998). Interactive effects of external manganese, the toxic metals copper and zinc, and light in controlling cellular manganese and growth in a coastal diatom. *Limnology and Oceanography*, *43*(7), 1467–1475.
Trimborn, S., Thoms, S., Bischof, K., & Beszteri, S. (2019). Susceptibility of two Southern Ocean phytoplankton key species to iron limitation and high light. *Frontiers in Marine Science*, *6*, 167.
Twining, B. S., Baines, S. B., Fisher, N. S., & Landry, M. R. (2004). Cellular iron contents of plankton during the Southern Ocean Iron Experiment (SOFeX). *Deep Sea Research Part I: Oceanographic Research Papers*, *51*(12), 1827–1850.
Twining, B. S., Baines, S. B., & Fisher, N. S. (2004). Element stoichiometries of individual plankton cells collected during the Southern Ocean Iron Experiment (SOFeX). *Limnology and Oceanography*, *49*(6), 2115–2128.
Twining, B. S., Rauschenberg, S., Morton, P. L., & Vogt, S. (2015). Metal contents of phytoplankton and labile particulate material in the North Atlantic Ocean. *Progress in Oceanography*, *137*, 261–283.

Wu, M., McCain, J. S. P., Rowland, E., Middag, R., Sandgren, M., Allen, A. E., & Bertrand, E. M. (2019). Manganese and iron deficiency in Southern Ocean Phaeocystis antarctica populations revealed through taxon-specific protein indicators. *Nature Communications*, *10*(1), 1–10.
